# Hypermethylated genome of a fish vertebrate iridovirus ISKNV plays important roles in viral infection

**DOI:** 10.1038/s42003-024-05919-x

**Published:** 2024-02-28

**Authors:** Mincong Liang, Weiqiang Pan, Yanlin You, Xiaowei Qin, Hualong Su, Zhipeng Zhan, Shaoping Weng, Changjun Guo, Jianguo He

**Affiliations:** 1 State Key Laboratory for Biocontrol, Southern Laboratory of Ocean Science and Engineering (Zhuhai), Guangdong Provincial Observation and Research Station for Marine Ranching of the Lingdingyang Bay, Guangdong Province Key Laboratory of Aquatic Economic Animals, School of Marine Sciences, Sun Yat-sen University, Guangzhou, Guangdong China; 2 Guangdong Laboratory for Lingnan Modern Agriculture, Guangzhou, Guangdong China

**Keywords:** DNA methylation, Viral immune evasion, Protein structure predictions

## Abstract

Iridoviruses are nucleocytoplasmic large dsDNA viruses that infect invertebrates and ectothermic vertebrates. The hypermethylated genome of vertebrate iridoviruses is unique among animal viruses. However, the map and function of iridovirus genomic methylation remain unknown. Herein, the methylated genome of Infectious spleen and kidney necrosis virus (ISKNV, a fish iridovirus), and its role in viral infection, are investigated. The methylation level of ISKNV is 23.44%. The hypermethylated genome is essential for ISKNV amplification, but there is no correlation between hypermethylation and viral gene expression. The hypomethylated ISKNV (obtained via 5-Azacytidine) activates a strong immunoreaction in vitro and reduces its pathogenicity in vivo. The unmethylated viral DNA can induce a stronger immunoreaction in vitro, whereas inactivated hypomethylated ISKNV can induce a stronger immunoreaction in vivo, suggesting ISKNV may evade from immune system by increasing its genome methylation level. Our work provides new insights into the role of genome methylation in viral infection.

## Abstract (web summary)

The hypermethylated genome of a fish vertebrate iridovirus is crucial for its infection and plays an important role in the virus’ ability to evade the host immune response.

## Introduction

DNA methylation is an epigenetic mechanism involving the transfer of a methyl group onto the C5 position of cytosine to form 5-methylcytosine, thereby frequently inhibiting gene expression and modifying the function of genes^1,2^. DNA methyltransferases (DNMT) promote the methylation process^3^. DNA methylation is directly involved in many important physiological and pathological processes, such as viral infection^4^. DNA containing unmethylated CpG sites can activate the innate immune response^5^. Many studies on viral genome methylation have mentioned that genome methylation is very important in the interaction between viruses and the host^6^. DNA methylation evades host immune surveillance to inhibit the viral gene transcription and replication, such as human T-cell leukemia virus type 1^7^, Epstein-Barr virus (EBV)^8^, hepatitis B virus (HBV)^9^, human papillomavirus (HPV)^10^, herpes simplex virus 1^11^, and adenovirus type 12^12^. Viral infection can affect the methylation level of some genes by regulating a variety of DNMTs to change the gene expression of the host, especially antiviral immune genes, such as Kaposi’s sarcoma herpesvirus^13^, EBV^14^, HBV^15^, and HPV^16^. These studies suggest that regulation of gene expression through DNA methylation may play a key role in the viral life cycle.

Iridovirids belong to the Nucleocytoviricota phylum, which comprises a group of viruses that have double-stranded DNA genomes and cytoplasmic phases that can infect both invertebrates and ectothermic vertebrates^17^. The *Iridoviridae* family is divided into two subfamilies: *Alphairidovirinae* includes *Lymphocystivirus*, *Ranavirus*, and *Megalocystivirus*, which infect vertebrate hosts (fish, amphibians, and reptiles), and *Betairidovirinae* includes *Iridovirus*, *Daphniairidovirus*, *Decapodiridovirus*, and *Chloriridovirus*, which infect invertebrate hosts^18^. Its characteristics of replication and translation can be roughly divided into three parts. The first stage DNA synthesis and early transcription takes place in the nucleus. Subsequently, DNA concatemer formation and late transcription occur in the cytoplasm. At last, the virion morphogenesis takes place in cytoplasmic assembly sites^17^. The genomes of vertebrate iridoviruses are unique in animal viruses, which have heavily methylated DNA due to a virus-encoded DNMT homolog^18,19^. Dawn B. Willis and Allan Granoff confirmed that more than 20% of the cytosine residues in frog virus 3 (FV3), a member of the genus *Ranavirus*, were methylated^20^. Schetter et al. suggested that nascent FV3 DNA in the nucleus is unmethylated or hypomethylated at an early stage and that viral DNA in the cytoplasm appears to be consistently hypermethylated^21^. Fish lymphocystis disease virus (FLDV), a member of the genus *Lymphocystivirus*, was also found to have a highly methylated genome by high-pressure liquid chromatography, nearest-neighbor analysis, and restriction endonucleases^22^. However, the sites, distribution characteristics, and functions of methylation in the iridovirus genome remain unknown. Infectious spleen and kidney necrosis virus (ISKNV), which belongs to the genus *Megalocytivirus* of the family *Iridoviridae*, has a genome with heavily methylated DNA^17^. It can infect more than 50 species of freshwater and marine fish, causing great economic losses during the past few decades^23^.

In this study, the genome-wide methylation landscape of ISKNV and the role of their genomic methylation was explored. Our work will help advance our understanding of vertebrate iridoviruses and improve our understanding of their immune escape mechanisms.

## Results

### Landscape of the hypermethylated genome in ISKNV virions

**Fig. 1:**
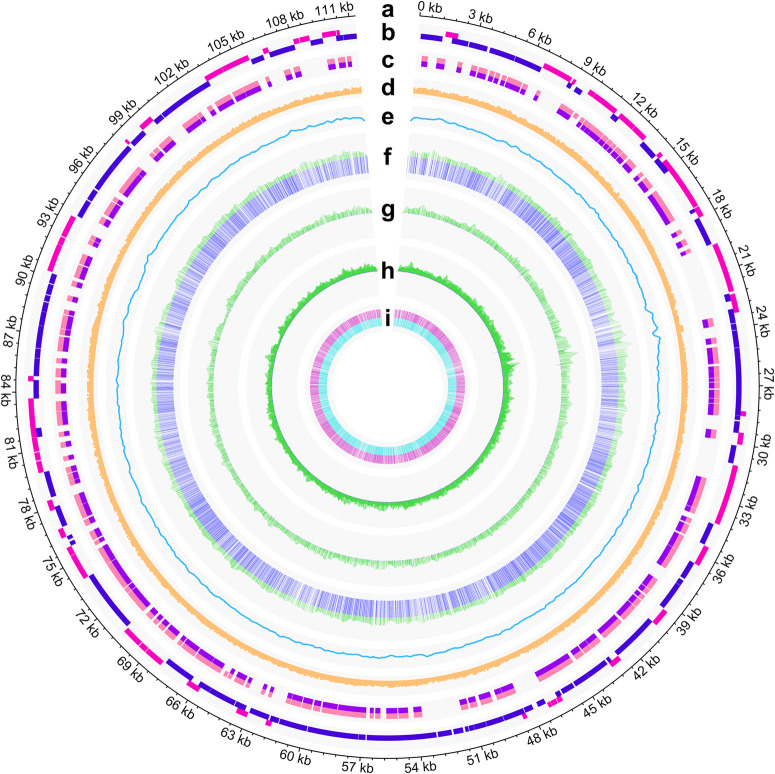
Hypermethylated genome-wide landscape of ISKNV. **a** The genome of ISKNV. **b** ORFs in the ISKNV genome, with dark pink representing ORFs in the R strand and dark purple representing those in the L strand. **c** CGIs in the ISKNV genome, with front pink representing CGIs in the R strand and front purple representing those in the L strand. **d** CG% per 100 bp in the ISKNV genome. **e** The sum of sequencing depth of WGBS at each position (*n* = 3). **f** Green represents the sum of methylated and unmethylated counts in WGBS, and dark blue represents the average methylation level of each CpG site (*n* = 3). **g** Green represents the sum of methylated and unmethylated counts in WGBS, and dark blue represents the average methylation level of each CHG site (*n* = 3). **h** Green represents the sum of methylated and unmethylated counts in WGBS, and dark blue represents the average methylation level of each CHH site (*n* = 3). **i** Carmine represents the average methylation level in the R strand, and cyan represents the average methylation level in the L strand of each CpG site (*n* = 3).

To obtain the site-by-site methylation landscape of the ISKNV genome, whole-genome bisulfite sequencing (WGBS) was performed on an ISKNV strain (OP896201.1). The ISKNV genome has a total length of 111,315 bp with 132 open reading frames (ORFs) and 275 CpG islands (CGIs), of which 50 ORFs and 139 CGIs were in the R strand (ISKNV genome, Fig. 1a; ORFs, Fig. 1b; CGIs, Fig. 1c; Supplementary Fig. 1 and Supplementary Data 1). The CG content in the genome was 54.77%, and the percentages of CpG (CG), CHG, CHH, and CHN (where H = A, T or G; N means there is no base at this site) in cytosine were 23.677%, 21.824%, 54.497%, and 0.002%, respectively (CG% per 100 bp, Fig. 1d and Supplementary Fig. 1).

**Fig. 2:**
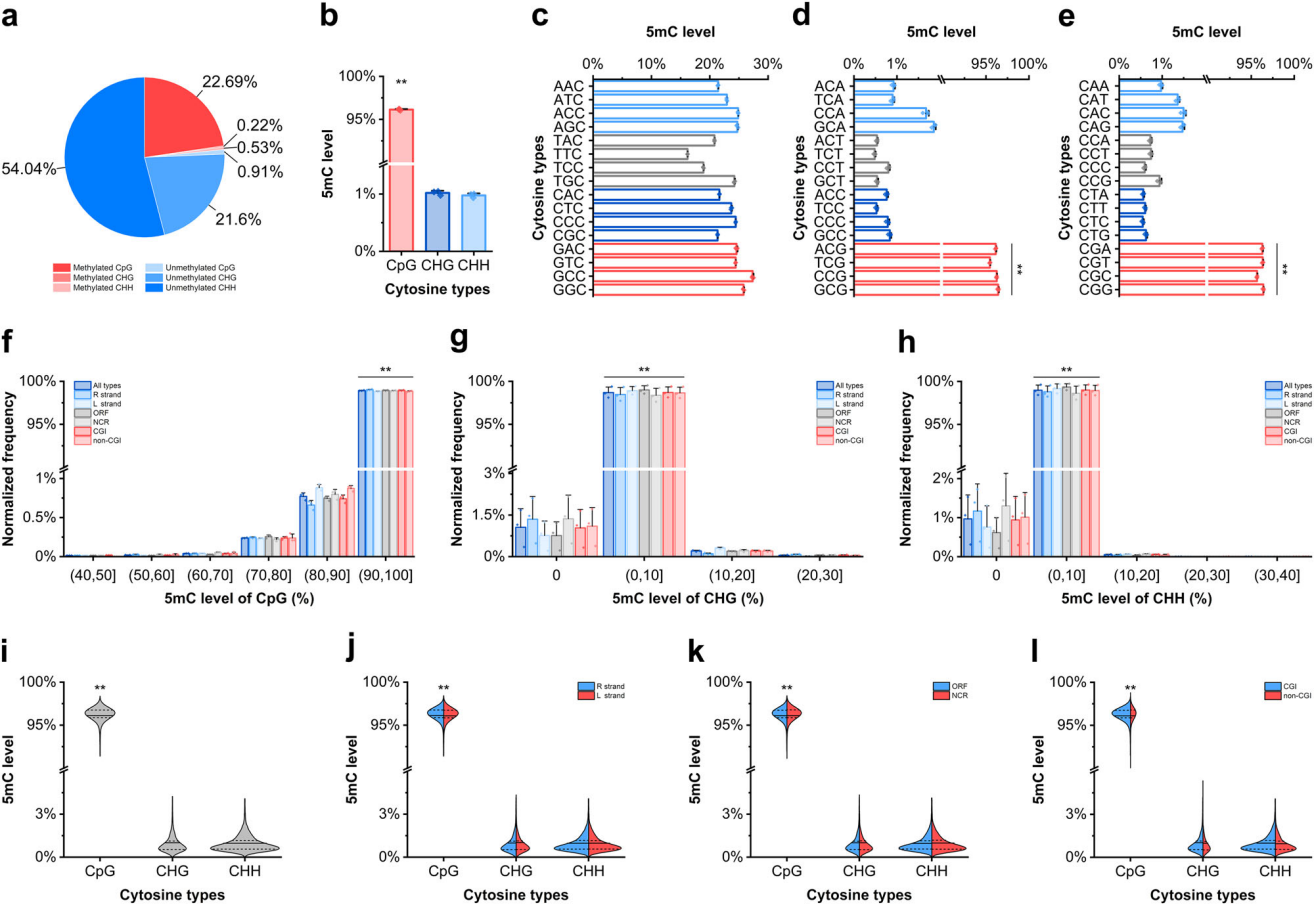
The ISKNV genome is highly methylated, and almost all CpG sites are highly methylated in all contexts. **a** The average percentage of methylated and unmethylated cytosines obtained from WGBS after extraction by Bismark. **b** Methylation levels of CpG, CHG, and CHH. **c** Methylation levels of cytosines distinguished by the following 2 bases in the 5’ region. **d** Methylation levels of cytosines distinguished by the following 1 base in the 5’ region and the following 1 base in the 3’ region. **e** Methylation levels of cytosines distinguished by the following 2 bases in the 3’ region. **f**–**h** Distribution of methylation levels of CpG, CHG, and CHH classified by different contexts. **i** The overall distribution of CpG, CHG, and CHH methylation levels in the ISKNV genome. **j** The overall distribution of CpG, CHG, and CHH methylation levels in the R and L strands. **k** The overall distribution of CpG, CHG, and CHH methylation levels in ORF and NCR. **l** The overall distribution of CpG, CHG, and CHH methylation levels in the CGI and non-CGI. Data are shown as the mean ± SD (*n* = 3) in (**b**–**h**). Solid lines represent the mean and the dashed lines represent the upper and lower quartiles in (**i**–**l**). Statistical significance is indicated by asterisks, with *p-value < 0.05 and **p-value < 0.01.

An average 2412.03x coverage site-by-site methylation landscape of the ISKNV genome was obtained from 3 duplicate samples (sequencing depth, Fig. 1e). Each cytosine was covered by WGBS except for each cytosine closest to 5’ and 3’ in the genome (Supplementary Data 2). The genome-wide methylation level was 23.44 ± 0.07%, and CpG sites contributed most of the methylation sites (Fig. 2a). The methylation levels of CpG, CHG, and CHH were 96.13 ± 0.07%, 1.02 ± 0.04%, and 0.98 ± 0.04%, respectively (5mC levels per site, Fig. 1f–i; total 5mC levels, Fig. 2b). The high methylation level in CpG sites was a surprising finding, and the effect of bases adjacent to cytosines on the methylation level of cytosines was explored. After classifying cytosines according to their nearby bases, it was found that the methylation level of the cytosines was independent of the 2 bases in their 5’ region (Fig. 2c). Regardless of the base type near the CpG site, the methylation level of all CpG sites was significantly higher than that of other sites (5mC distinguished by the following 1 base in the 5’ region and the following 1 base in the 3’ region, Fig. 2d; 5mC distinguished by the following 2 bases in the 3’ region, Fig. 2e).

The methylation of cytosines in different regions of the viral genome was further investigated, including the R strand, L strand, CGI, non-CGI, ORF, and noncoding regions (NCR). The statistical results showed that the distribution of cytosine methylation levels was consistent with the distribution in the whole genome regardless of the kind of region in the genome. The methylation levels of most CpG sites were more than 90%, while the methylation levels of most CHG and CHH sites were less than 10% (normalized frequency of 5mC levels, Fig. 2f–h). The distribution of hypermethylated CpG sites and hypomethylated CHG and CHH sites was concentrated in all kinds of regions of the viral genome. The CGI regions contained more cytosines than the non-CGI regions (distribution of 5mC sites in the genome, Fig. 2i; distribution of 5mC sites in different kinds of regions, Fig. 2j–l).

The overall higher level of CpG methylation in the ISKNV genome is due to the higher level of methylation at each CpG site rather than the existence of a mixture of CpG sites with low and high levels of methylation. The results suggested that the ISKNV genome and its CpG sites were hypermethylated.

### Molecular experiments demonstrated that the genome of ISKNV is heavily methylated

**Fig. 3:**
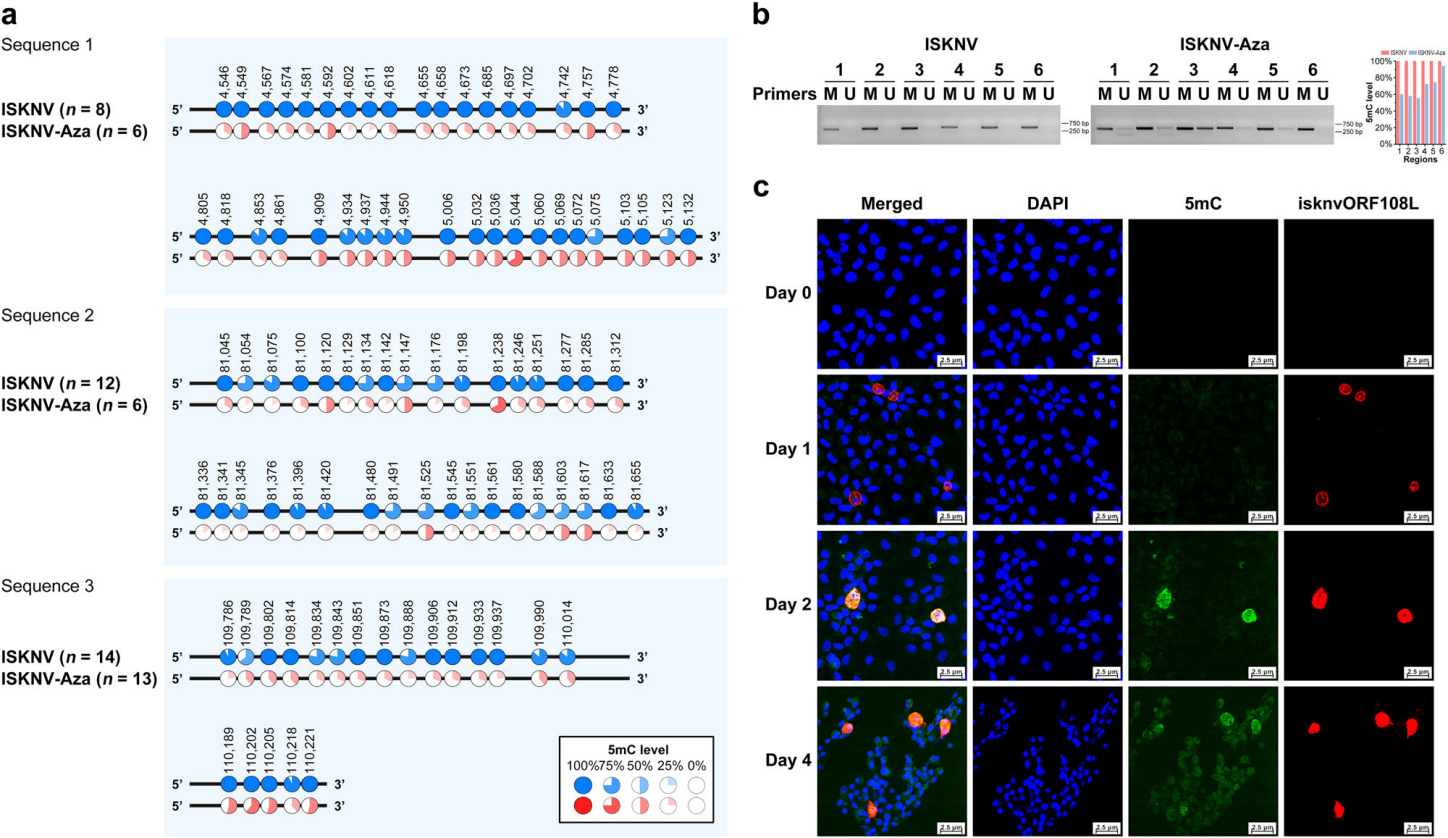
Molecular experiments demonstrated that the ISKNV genome is highly methylated. **a** The BSP results of 3 regions in the ISKNV and ISKNV-Aza genomes demonstrate the methylation level of each CpG site in these regions. **b** The MSP results of 6 regions in the ISKNV and ISKNV-Aza genomes. The histogram shows methylation levels in different regions of the viral genome obtained by MSP, calculated as “gray value from M primers / (gray value from M primers + gray value from U primers)”. **c** Immunofluorescence of MFF-1 cells infected with ISKNV (MOI = 1) at 0, 1, 2, and 4 days; blue represents nuclei, green represents DNA containing 5mC, and red represents isknvORF108L. The scale bars in the figure represent 2.5 μm. In the figure, “M Primers” indicates methylated primers, and “U Primers” indicates unmethylated primers.

To further verify the genome-wide methylation sequencing data, methylation-specific PCR (MSP) and bisulfite sequencing PCR (BSP) were performed on the DNA derived from the virions. Six randomly selected sequences from the ISKNV genome were investigated using MSP, and three of them were investigated using BSP. Both BSP and MSP demonstrated the presence of hypermethylated CpG sites in the genome of ISKNV (BSP, Fig. 3a; MSP, Fig. 3b).

In addition, the reduction of CpG methylation level on the virions from mandarin fish fry (MFF-1) cells infected with ISKNV by adding 5-Azacytidine, which is a commonly used DNA methylation inhibitor^24^ and can reduce the intracellular DNA methylation level by inhibiting DNMT activity^25^, was detected by using the same primers in MSP and BSP. The results of BSP showed that the methylation level of the hypermethylated CpG sites in the ISKNV genome was significantly decreased after 5-Azacytidine treatment (Fig. 3a). MSP further showed that only methylated bands could be amplified in ISKNV by using methylated primers, and unmethylated bands could be amplified by unmethylated primers after the addition of 5-Azacytidine (Fig. 3b). Immunofluorescence results demonstrated that the intracellular DNA containing 5mC increased with the proliferation of ISKNV in MFF-1 cells (Fig. 3c). The hypomethylated virions obtained after the addition of 5-Azacytidine were named ISKNV-Aza. These results indicated that the genome of ISKNV is heavily methylated and that 5-Azacytidine could reduce the methylation level.

### Hypermethylation of the genome plays an essential role in ISKNV replication

**Fig. 4:**
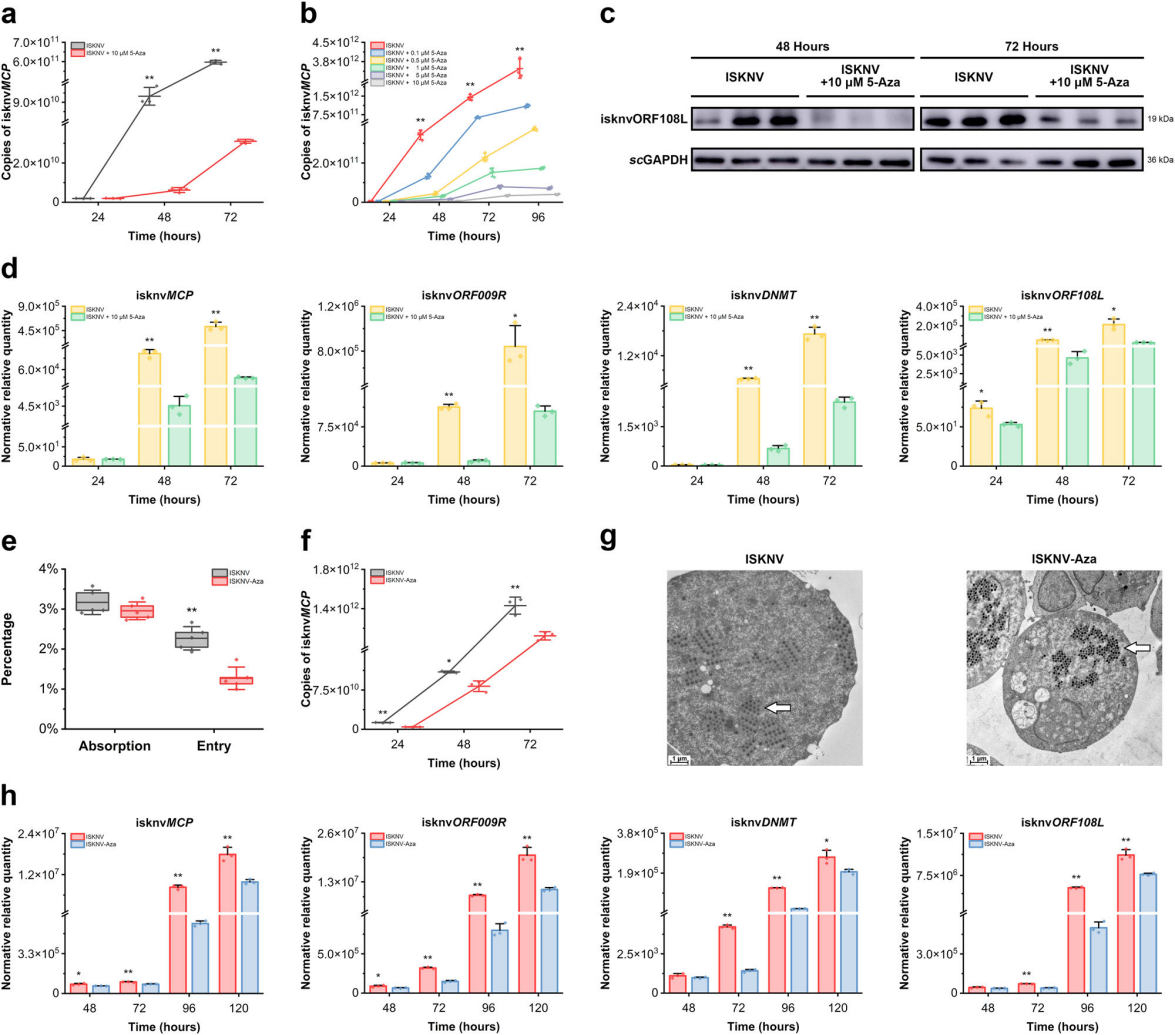
Hypermethylation of the ISKNV genome is important for its life cycle in its host. **a**–**d** After infecting MFF-1 cells with ISKNV (MOI = 1) with different concentrations of 5-Azacytidine. **a**, **b** Copies of isknv*MCP* were determined by qPCR at 24, 48, 72, and 96 h. **c** Expression of isknvORF108L was determined by western blotting at 48 and 72 h. **d** Relative mRNA levels of isknv*MCP*, isknv*ORF009R*, isknv*DNMT*, and isknv*ORF108L* were measured by RT-qPCR at 24, 48, and 72 h. **e** Adsorption and entry efficiency of ISKNV and ISKNV-Aza (*n* = 5). After infecting MFF-1 cells with 1 × 10^9^ copies of ISKNV or ISKNV-Aza per well, the cells were placed at 4 °C for 1 h, and the total DNA was extracted after discarding the culture medium to test the adsorption efficiency by qPCR. Cells infected with the same copy number of viruses were placed at 27 °C for 4 h, the culture medium was discarded, and the total DNA was extracted to detect the entry efficiency. The box limited is from the upper to the lower quartiles. The center lines represent the mean and the whiskers represent the SD. **f**–**h** After infecting MFF-1 cells with ISKNV and ISKNV-Aza at 1 × 10^7^ TCID_50_/mL. **f** Copies of isknv*MCP* were determined by qPCR at 24, 48, and 72 h. **g** Virus lattice formation in MFF-1 cells was observed by transmission electron microscopy at 72 h. The scale bars in the figure represent 1 μm. **h** Relative mRNA levels of isknv*MCP*, isknv*ORF009R*, isknv*DNMT*, and isknv*ORF108L* were measured by RT-qPCR at 48, 72, 96, and 120 h. In the figure, “5-Aza” indicates 5-Azacytidine. Data are shown as the mean ± SD (*n* = 3). Statistical significance is indicated by asterisks, with *p-value < 0.05 and **p-value < 0.01.

While exploring whether 5-Azacytidine could reduce the 5mC levels of ISKNV virions, it was found that the onset of symptoms in MFF-1 cells after infection with ISKNV was significantly delayed by the addition of 5-Azacytidine (Supplementary Fig. 2). The cell activity experiment proved that 5-Azacytidine at low concentrations did not affect cell activity (Supplementary Fig. 3). Therefore, the effect of 5-Azacytidine on the duplication, transcription, and translation of ISKNV was investigated. Adding 5-Azacytidine at the same time as infecting MFF-1 cells with ISKNV significantly inhibited the viral DNA (copies of isknv*MCP*, Fig. 4a, b), mRNAs (expressions of isknv*MCP*, isknv*ORF009R*, isknv*DNMT*, and isknv*ORF108L* genes, Fig. 4d), and protein (isknvORF108L, Fig. 4c) levels of ISKNV, and those inhibitions were significantly enhanced with increasing 5-Azacytidine concentration. Furthermore, the adsorption ability of the hypomethylated virions ISKNV-Aza to MFF-1 cells was not decreased, while ISKNV-Aza’s invasion ability was decreased, but it did not affect lattice formation in MFF-1 cells (the ability of adsorption and invasion, Fig. 4e; viral lattice in cells, Fig. 4g). However, the duplication and transcription of ISKNV-Aza were significantly inhibited compared to those of ISKNV (copies of isknv*MCP*, Fig. 4f; expressions of isknv*MCP*, isknv*ORF009R*, isknv*DNMT*, and isknv*ORF108L* genes, Fig. 4h). The replication level of ISKNV was higher than that of ISKNV-Aza from 24 to 72 h after infection, with differences of 2.9, 1.3, and 1.3 times at 24, 48, and 72 h, respectively (Fig. 4f). The expression levels of the four target genes of ISKNV were higher than those of ISKNV-Aza from 72 to 120 h after infection. The differences in isknv*MCP*, isknv*ORF008R*, isknv*DNMT*, and isknv*ORF108L* at 120 h were 1.8, 1.8, 1.4, and 1.5 times, respectively (Fig. 4h). These results suggested that hypermethylation of the ISKNV genome played important roles in the replication of ISKNV.

### The hypermethylation of the ISKNV genome was not associated with the expression of viral genes

**Fig. 5:**
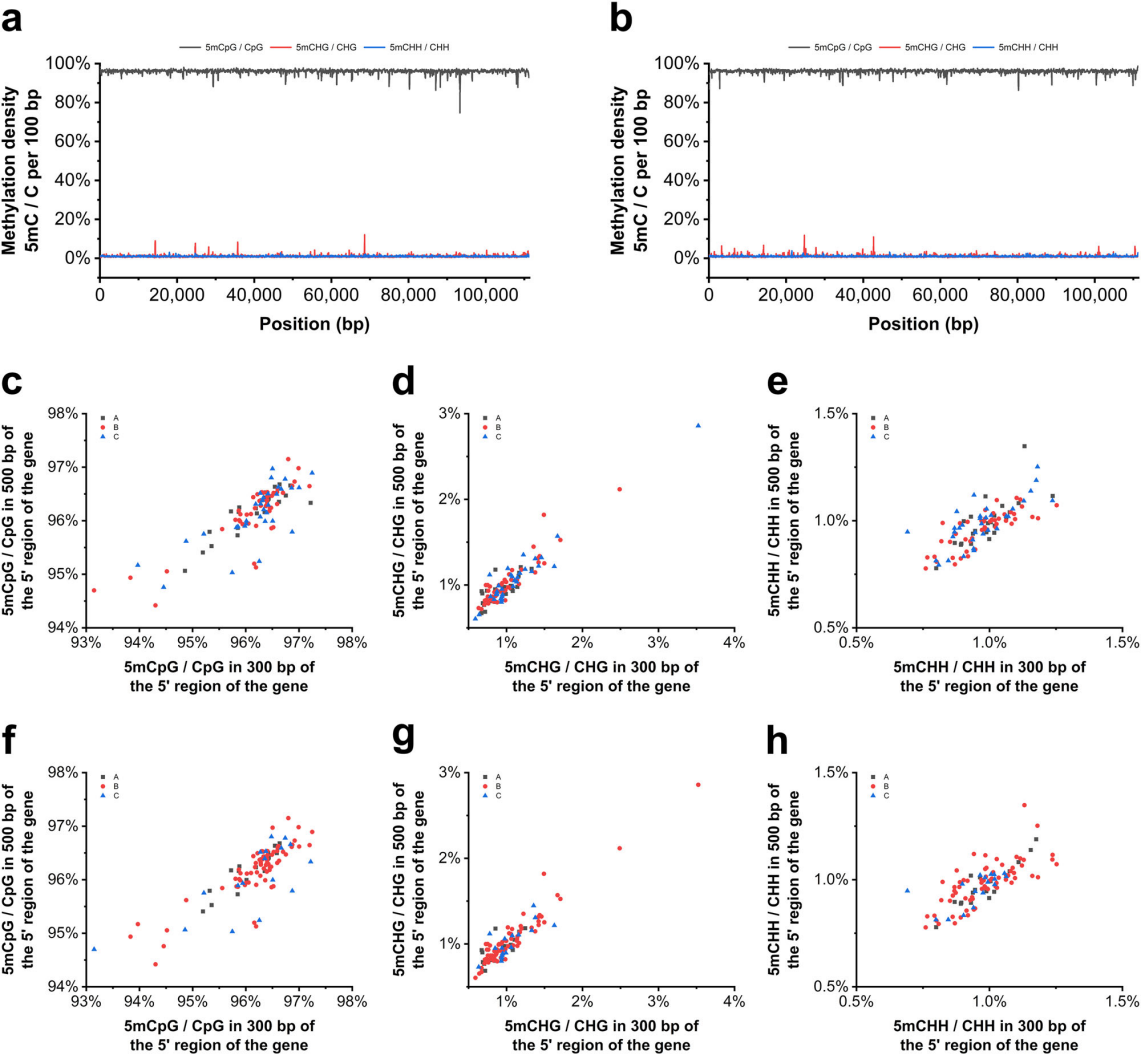
Hypermethylation of the ISKNV genome is not associated with gene expression. **a** Methylation density per 100 bp in the R strand of the ISKNV genome. **b** Methylation density per 100 bp in the L strand of the ISKNV genome. **c**–**e** CpG, CHG, and CHH methylation density of 300 bp and 500 bp in the 5’ region of ORFs of different expression types in vitro. **f**–**h** CpG, CHG, and CHH methylation density of 300 bp and 500 bp in the 5’ region of ORFs of different expression types in vivo.

Methylation is generally thought to be associated with gene expression^4,26^. To explore whether the heavily methylated genome was associated with the expression of ISKNV viral genes, the genome-wide methylation density per 100 bp for three types of cytosines was obtained. Regardless of the R or L strand, the methylation density of the three types of cytosines was stable, and there was no large fluctuation. 5mCpG / CpG fluctuates at approximately 95%, while 5mCHG / CHG and 5mCHH / CHH fluctuate at approximately 1% (methylation density in R and L strand, respectively, Fig. 5a, b).

He, J. et al.^27^ divided ISKNV genes into the following three groups based on their expression in vivo and in vitro: Cluster A, ORFs with low expression in the early stage and moderate expression in the late stage; Cluster B, ORFs expressed as early as 1 h postinfection with a certain level; and Cluster C, ORFs with low expression in the early stage and high expression in the late stage. The methylation density of 300 bp and 500 bp in the 5’ region of ORFs was obtained and divided according to the above classification to explore whether the expression of viral genes was related to the methylation levels of the promoter regions. The results showed that the methylation densities of the three cytosines in the 5’ region of the ORFs were independent of their expression regardless of whether the ORFs were divided in vitro or in vivo (methylation densities when the ORFs were divided in vitro, Fig. 5c–e; methylation densities when the ORFs were divided in vivo, Fig. 5f–h). These results suggested that the rule that higher levels of methylation are associated with lower levels of gene expression does not apply in ISKNV.

### The heavy methylation of the ISKNV genome might involve in viral immune escape

**Fig. 6:**
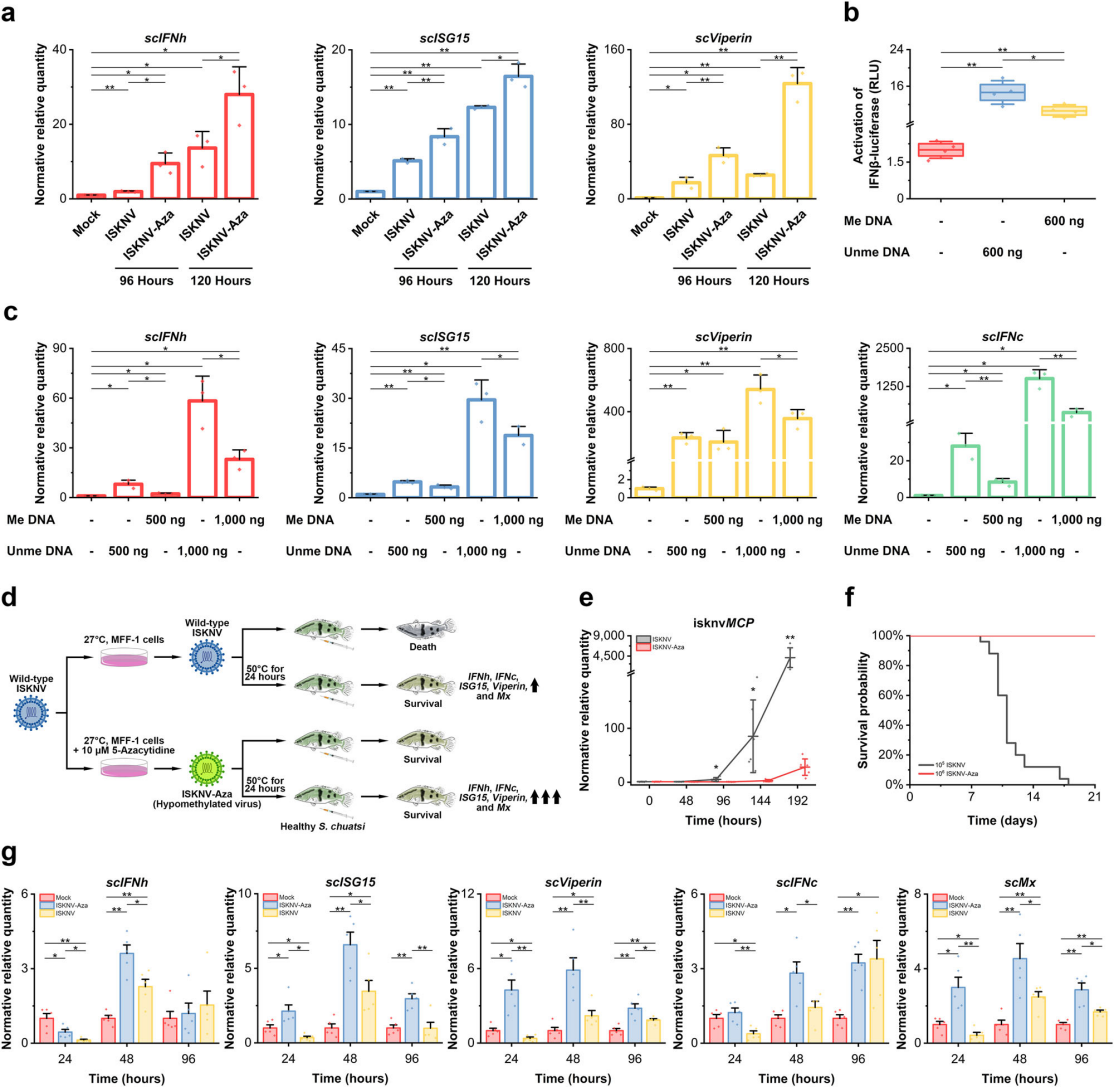
The highly methylated genome of ISKNV might be involved in its immune escape. **a** Relative mRNA levels of *scIFNh*, *scISG15*, and *scViperin* were measured by RT-qPCR after infecting MFF-1 cells with ISKNV and ISKNV-Aza at 1 × 10^7^ TCID_50_/mL at 96 and 120 h (*n* = 3). **b** Activation of interferon reporter genes after transfection with methylated or unmethylated fragments was measured by dual luciferase assays at 36 h (*n* = 4). The box limited is from the upper to the lower quartiles. **c** Relative mRNA levels of *scIFNh*, *scISG15*, *scViperin*, and *scIFNc* were measured by RT-qPCR after transfection with methylated or unmethylated fragments at 48 h (*n* = 3). **d** Schematic diagram of the experimental procedure in vivo. **e** Relative mRNA levels of isknv*MCP* from fish spleen measured by RT-qPCR after intraperitoneal injection of 1 × 10^8^ copies of ISKNV or ISKNV-Aza at 48, 96, 144, and 192 h (*n* = 6). **f** Survival curves of *S. chuatsi* after intraperitoneal injection of 1 × 10^5^ copies of ISKNV or 1 × 10^6^ copies of ISKNV-Aza (*n* = 25). **g** Relative mRNA levels of *scIFNh*, *scISG15*, *scViperin*, *scIFNc*, and *scMX* from fish spleen measured by RT-qPCR after intraperitoneal injection of 2 × 10^11^ copies of inactivated ISKNV or inactivated ISKNV-Aza at 24, 48, and 96 h (*n* = 5). In the figure, “Me DNA” indicates methylated DNA, and “Unme DNA” indicates unmethylated DNA. Data are shown as the mean ± SD. Statistical significance is indicated by asterisks, with *p-value < 0.05 and **p-value < 0.01.

To explore whether the highly methylated CpG sites in the ISKNV genome were involved in viral immune escape, the expression of immune-related genes (*IFN*s and *ISG*s) was determined in cells infected with ISKNV or ISKNV-Aza. Compared with ISKNV, hypomethylated ISKNV-Aza could activate a higher immune response in MFF-1 cells (expressions of *scIFNh*, *scISG15*, and *scViperin* genes, Fig. 6a). On the 96 or 120 h after infection, ISKNV-Aza increased the activation of *scIFNh* by 2.1 to 5.7 times, *scViperin* by 2.7 to 4.9 times, and *scISG15* by 34 to 63% compared with ISKNV. To demonstrate that differences in methylation in the viral genome contribute to the different activation of immune responses, a 388 bp DNA fragment containing various parts of isknv*ORF015L* and isknv*ORF016R* from purified viral DNA was digested by double restriction enzyme digestion, and the methylated and unmethylated DNA fragments were obtained after PCR and CpG methyltransferase (M. SssI) treatment. Dual-luciferase assay and qPCR demonstrated that the unmethylated DNA fragment could activate a higher immune response in MFF-1 cells than the methylated DNA fragment in a dose-dependent manner (activation of IFNβ-luciferase, Fig. 6b; expressions of *scIFNh*, *scISG15*, *scViperin*, and *scIFNc* genes, Fig. 6c). In the dual-luciferase assay, unmethylated DNA showed an approximately 1.4-fold increase in interferon activation in vitro compared to methylated DNA (Fig. 6b). At 48 h after transfection, compared with methylated DNA, unmethylated DNA showed a 2.5-to-3.6-fold increase in *scIFNh* activation, a 3.3-to-3.8-fold increase in *scIFNc* activation, a 13 to 52% increase in *scViperin* activation, and a 47 to 57% increase in *scISG15* activation (Fig. 6c).

To further verify the relationship between the level of genomic methylation and innate immunity, different viruses were injected into mandarin fish (*Siniperca chuatsi*) to carry out a series of experiments (Fig. 6d). However, unlike in vitro experiments, compared with wild-type ISKNV, which could proliferate continuously in vivo, hypomethylated ISKNV-Aza could hardly reproduce in *S. chuatsi* (copies of isknv*MCP*, Fig. 6e). After 192 h of infection, isknv*MCP* of ISKNV was expressed more than 147 times in *S. chuatsi* compared with ISKNV-Aza. The survival rate experiment showed that compared with wild-type ISKNV with a 0% survival rate, *S. chuatsi* could still maintain a 100% survival rate even after injection of a higher number of copies of hypomethylated ISKNV-Aza (Fig. 6f).

To further confirm whether the hypomethylated genomic DNA can activate innate immunity in vivo and to verify whether the reduced transcription level of ISKNV-Aza in vivo is activated due to innate immunity, ISKNV and ISKNV-Aza were inactivated at 50 °C for 24 h^28^, and cell experiments showed that the virus had lost its infectivity and could not replicate and transcribe in vitro (Supplementary Fig. 4). An equal number of copies of inactivated ISKNV or inactivated ISKNV-Aza were intraperitoneally injected into *S. chuatsi*. At 24 and 48 h after injection, the expression levels of *scIFNh* and *scIFNc* in the fish injected with inactivated ISKNV-Aza were significantly higher than those injected with inactivated ISKNV, while there was no significant difference between the two groups at 96 h, but the expression levels of *scIFNc* were significantly higher than those in the control group. This was supported by the expression levels of other genes related to the innate immune system. At all times, the expressions of *scISG15*, *scViperin*, and *scMX* were significantly higher in the fish injected with inactivated ISKNV-Aza than in those injected with inactivated ISKNV (expressions of *scIFNh*, *scISG15*, *scViperin*, *scIFNc*, and *scMX* genes, Fig. 6g). At 24 h after injection, inactivated ISKNV-Aza increased the activation of *scIFNh* by 3.5 times, *scISG15* by 6.1 times, *scViperin* by 11.3 times, *scIFNc* by 3.2 times, and *scMX* by 7.1 times in vivo compared with inactivated ISKNV. At 48 h after injection, inactivated ISKNV-Aza increased the activation of *scIFNh* by 1.6 times, *scISG15* by 1.9 times, *scViperin* by 2.7 times, *scIFNc* by 2 times, and *scMX* by 1.8 times in vivo compared with inactivated ISKNV. At 96 h after injection, inactivated ISKNV-Aza increased the activation of *scISG15* by 2.3 times, *scViperin* by 1.5 times, and *scMX* by 1.7 times in vivo compared with inactivated ISKNV. These results suggest that hypomethylated DNA in the viral genome can significantly activate the innate immune system in the host. These findings indicate that the highly methylated ISKNV genome could lead to lower immune activation and might involve in some immune escape processes.

### IsknvDNMT has the ability to de novo methylate DNA similar to DNMT3

**Fig. 7:**
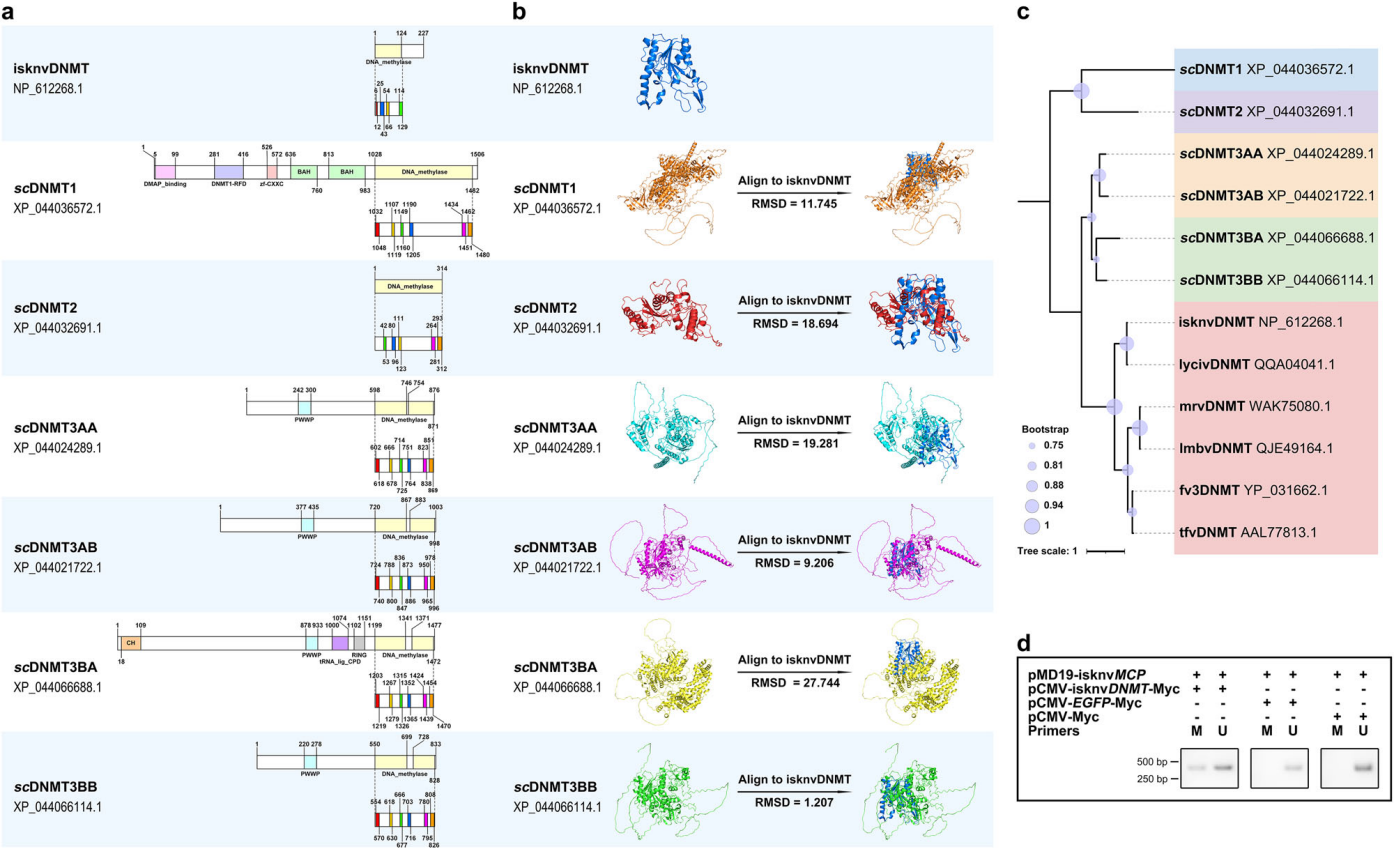
IsknvDNMT has the ability to de novo methylate DNA similar to DNMT3. **a** Protein structure partitioning and motif identification in the DNA methylation catalytic domain for isknvDNMT and *sc*DNMTs. Red, yellow, green, blue, purple, and orange represent motifs I, IV, VI, VIII, IX, and X, respectively. **b** Protein structure prediction and protein structure alignment for isknvDNMT and *sc*DNMTs. **c** Phylogenetic trees based on isknvDNMT, *sc*DNMTs, and other iridovirus DNMT proteins. **d** pMD19-isknv*MCP* was cotransfected into HEK293T cells with pCMV-isknv*DNMT*-Myc, pCMV-*EGFP*-Myc, or pCMV-Myc, and the methylation level of pMD19-isknv*MCP* was measured by MSP at 48 h. In the figure, “M Primers” indicates methylated primers, and “U Primers” indicates unmethylated primers.

DNMTs promote the methylation process^3^. Like other iridoviruses, ISKNV encodes a protein that functions as a methyltransferase, named isknvDNMT. Domain analysis and protein structure prediction for the isknvDNMT and 6 different types of DNMT obtained from NCBI for its host, *S. chuatsi*, were performed. A more detailed internal division of the DNA methylation catalytic domain was also performed. Domain analysis showed that isknvDNMT had the same DNA methylation catalytic domain as other *sc*DNMTs, but shorter (Fig. 7a). Although the sequence of sites within the DNA methylation catalytic domain of isknvDNMT was inconsistent and the IX and X motifs were missing, the alignment of the high-precision models obtained by AlphaFold prediction showed that *sc*DNMT3BB was most similar to isknvDNMT in protein structure (RMSD = 1.207) (protein structure alignment, Fig. 7b and Supplementary Fig. 5). The results of the protein evolutionary tree also helped to prove this (Fig. 7c). Unlike DNMT1, which functions to maintain methylation, DNMT3 has been reported to function in de novo methylation. Since the results of protein structure alignment and the protein evolutionary tree both suggest that isknvDNMT is more closely related to *sc*DNMT3BB, isknvDNMT may also have the ability to perform de novo methylation. To verify this hypothesis, different plasmids were transfected into cells, and the MSP results showed that isknvDNMT could methylate exogenous plasmids (Fig. 7d). This result demonstrated that isknvDNMT can de novo methylate foreign DNA fragments such as DNMT3.

## Discussion

DNA methylation has been widely studied as an important epigenetic pathway. In organisms, DNA methylation is essential for normal development and is associated with many key processes, such as gene regulation, genomic imprinting, transposable element repression, immune response, and virus infection^4^. Many studies have explored the form and role of cytosine methylation in animal DNA viruses^6^, and most of these studies have focused on the effect of methylation on the gene expression of the virus or host. Unlike the approximate 5% methylation level in humans^29^ and *Arabidopsis*^30^, the methylation level of viruses is more variable, with some viruses having different methylation statuses during active replication and latency. The DNA methylation levels of adenovirus type 2 and type 12 were 0.04 and 0.06%, respectively, while the DNA methylation level of hamster cells transformed by adenovirus type 12 was approximately 3%^31^. The proportion of methylated cytosine in the DNA of adeno-associated virus type 2 (AAV2) virions was between 0 and 1.7%, while the average methylation level of each CpG site in the chromosome-integrated AAV2 genome was 76%^32^. The same phenomenon was observed in HPVs^33,34^ and polyoma virus^35^. However, we found a methylation level of 23.44% for ISKNV, which was similar to the previously reported for FV3 and FLDV. In addition, the landscape of ISKNV genomic methylation profiles was further characterized. In the ISKNV genome, the methylation levels of CpG, CHG, and CHH sites were 96.13%, 1.02%, and 0.98%, respectively. Therefore, we can conclude that the genome of ISKNV is heavily methylated, and that the methylation is almost exclusively contributed by CpG sites. Almost all CpG sites in the ISKNV genome are heavily methylated. The hypermethylation of CpG sites is not affected by the location of the site in the genome or by the bases in the vicinity of the site.

Methylation is usually associated with gene expression. In previous studies, DNA methylation may affect gene transcription in two ways: DNA methylation hinders the binding of transcription proteins to DNA^36^, and methylated DNA is bound to proteins called methyl-CpG-binding domain proteins (MBDS) to form compact, inactive chromatin. This phenomenon in which gene expression decreases with increasing DNA methylation levels exists in a variety of organisms, including viruses, that need to use the host to complete their life cycle. For example, EBV can control the expression of its genes by regulating the methylation level of its genome^37^. However, for ISKNV, which has a highly methylated genome and whose methylation density fluctuates little in the genome, DNA methylation appears to be independent of its gene expression. Classification of ISKNV ORFs by expression signature did not reveal a clear association with methylation levels. This indicates that ISKNV gene expression levels are not affected by methylation levels in the DNA regions, which is inconsistent with other previous studies that gene methylation levels are negatively correlated with gene expression. A similar phenomenon was also demonstrated in FV3. Thompson, J. P. et al.^38^ have found that methylation of immediate-early genomic promoters in FV3 did not affect its transcription. In earlier studies, Thompson, J. P. et al.^39^ found that FV3-infected cells were able to transcribe highly methylated DNA, while virus-induced trans-acting proteins may alter the host’s RNA polymerase II, or methylated DNA template, to allow transcription from methylated promoters. These results suggest that virus-induced trans-acting proteins may be able to overcome the transcriptional inhibition of methylation, this phenomenon is also likely to be present in ISKNV. It also suggests that the highly methylated genome of ISKNV may have other more important roles.

The include immune escape mechanisms of animal viruses mainly include the following three types: antigen mutation of the virus, escape from the neutralization and blocking effect of the existing antibodies; the intracellular virus is in a dormant state in the cell to escape the attack of cellular and humoral immunity; and viruses antagonize, block, and inhibit the body’s immune response through their structural and nonstructural products. Most of the current reports on ISKNV-associated immune escape have focused on the latter mechanism. ISKNV vSOCS inhibits IFN-α-induced Jak-Stat signaling to evade IFN-antiviral immunity^40^. The ISKNV ORF124L protein inhibits TNF-α-induced NF-κB signaling through its interaction with IKKβ^41^. The ISKNV vTRAF protein can induce apoptosis by interacting with TRADD^42^. This study revealed another possible immune escape pathway of ISKNV, which inhibits host immune responses through hypermethylated DNA to evade the recognition of pattern recognition receptors on foreign DNA. For viruses that need to expose their DNA to the cytosolic environment for a long time, it is inevitable that their DNA is recognized by innate pattern recognition receptors. However, according to previous reports, congenital pattern recognition receptors such as cGAS and the TLR family seem to be sensitive only to unmethylated CpG sequences, and methylated CpG sequences can reduce binding to the receptor or even not bind at all^43–47^. This could explain why hypomethylated ISKNV and unmethylated ISKNV could activate stronger immune responses in MFF-1 cells and why hypomethylated ISKNV possessed lower replication and transcription levels. The relative mRNA levels of isknv*MCP* in vivo supported this conclusion. To further rule out the effects of viral proteins on the host immune system and focus on the effects of viral genomic DNA on the host immune system, ISKNV and ISKNV-Aza were inactivated by using high temperatures. The results showed that the activation of the host immune system of the inactivated hypomethylated virus was always higher than that of the inactivated wild-type virus. This effect was immediately evident in the expression of interferon 24 h after injection and was sustained at 48 h. Although interferon expression levels tended to be consistent between the two groups 96 h after injection, the expression levels of downstream interferon genes demonstrated that this activation effect persisted. This result suggests that the methylation level of the viral genome is directly involved in the fight against the host immune system, rather than indirectly regulating genes through methylation.

This hypothesis may account for the inhibition of the replication, transcription, and translation of ISKNV by adding 5-Azacytidine, which is caused by the fact that the newborn DNA cannot be methylated. Large quantities of foreign DNA can activate the innate immune system of the host cell, thereby suppressing the virus. The same situation exists in hypomethylated ISKNV, which has weaker entry, transcription, and replication abilities compared to wild-type ISKNV. This finding suggests a different immune escape mechanism that may also exist in other large cytoplasmic dsDNA viruses and warrants further study of its specific molecular mechanisms.

The DNMT1 enzyme plays a major role in maintaining methylation, and hemimethylated DNA strands are methylated by the DNMT1 enzyme after DNA replication. DNMT3A and 3B are involved in the de novo methylation of cytosine residues. DNMT3L is a nuclear protein that does not contain the methyltransferase active site motif, so it cannot actively add methyl groups to cytosine residues. DNMT2 is structurally and functionally distinct from other DNMTs because it does not possess an N-terminal regulatory domain and is unable to catalyze de novo or maintain the methylation process^48^. DNMT is essential for biological development. Since DNMT is required for mammalian cell development, DNMT1 knockout (*DNMT1*^-/-^) mouse embryos cannot survive, while DNMT3A knockout (*DNMT3A*^-/-^) mice show partial defects. DNMT3B knockout (*DNMT3B*^-/-^) results in embryonic lethality similar to that of the *DNMT1*^-/-^ knockout mouse model^49^. DNMT is also essential for Iridovirus. In an earlier study, Whitley, D. S. et al.^50^ used RNA interference technology to demonstrate that DNMT was essential for FV3 replication. The knockdown of DNMT resulted in a greater than 95% reduction in virus production and a significant reduction in virus particle formation. This is consistent with our attempt to obtain ISKNV mutant by replacing DNMT with EGFP gene using homologous recombination, where we observed that the virus was almost unable to replicate and form mature virions (data unpresented due to negative results). We hypothesized that this was related to the lack of methylation protection of newly generated viral DNA against the innate immune attack of the host. In this study, we demonstrate that isknvDNMT has a DNMT3-like de novo methylation ability, although isknvDNMT lacks the IX and X motifs in the DNA methylation catalytic domain. According to reports in the literature, motif IX maintains the stability of substrate binding sites, and motif X plays a role in the formation of AdoMet binding sites^48^.

**Fig. 8:**
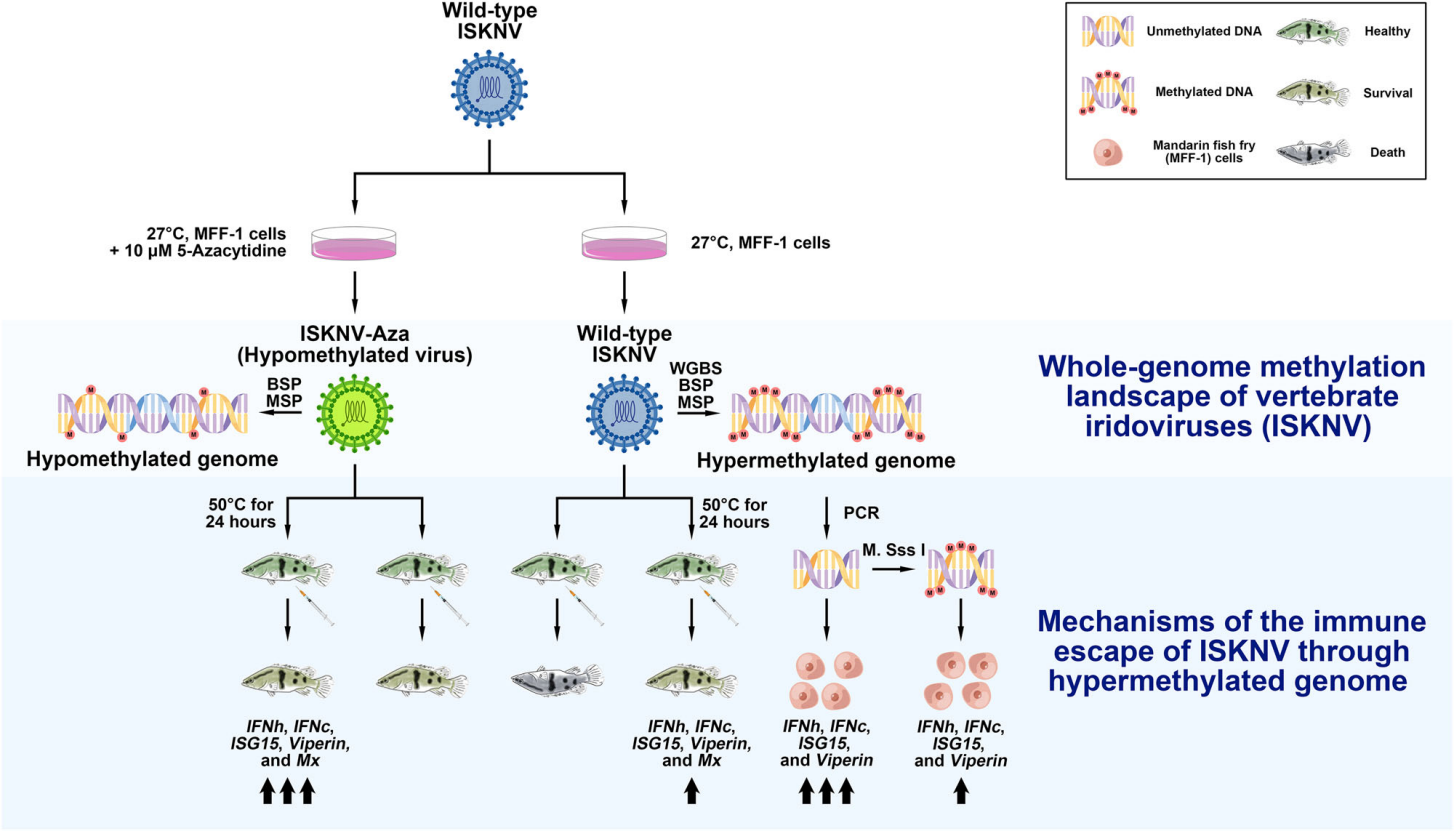
Schematic representation of a potential immune escape mechanism of ISKNV through the hypermethylated genome. The genomic methylation landscape of wild-type ISKNV was interpreted by WGBS and confirmed by MSP and BSP. The unmethylated viral DNA could induce a stronger immunoreaction in vitro. The hypomethylated ISKNV (obtained via 5-Azacytidine) activated a strong immunoreaction in vitro and reduced its pathogenicity in vivo, whereas inactivated hypomethylated ISKNV could induce a stronger immunoreaction in vivo, suggesting ISKNV may evade from the immune system by increasing its genome methylation level.

In conclusion, our work has obtained a hypermethylated genome-wide landscape of ISKNV, explaining the role of the highly methylated genome and providing new insights into the involvement of viral genome methylation in immune escape (Fig. 8) and may aid in the study of the mechanisms of host-virus interactions and epigenetics in viruses.

## Materials and Methods

### Fish and cells

Mandarin fish (*Siniperca chuatsi*), weighing 50 ± 10 g and less than 1 year old, were collected from a commercial fish farm in Foshan, Guangdong Province, China. Twenty-five fish were maintained in a 100 L glass aquarium tank with continuous ventilation at 27 ± 2 °C before and during the experiment. The fish were kept in the same environment for 7 days, and PCR tests were conducted to confirm that they were not infected with ISKNV, Mandarin fish ranavirus (MRV), and Siniperca chuatsi rhabdovirus (SCRV) before the experiment began. The primers used are listed in Supplementary Data 3. Meanwhile, the fish were fed a meal equal to 5% of their body weight, once a day, and 30% of the water in the tank was replaced with fresh water with continuous ventilation at the same temperature every day. Mandarin fish fry (MFF-1) cells were cultured at 27 °C and 5% CO_2_ using Dulbecco’s modified Eagle medium (DMEM, Gibco, USA) containing 10% fetal bovine serum (FBS, HyClone, USA)^51^.

### Viruses

The wild-type ISKNV strain for whole-genome bisulfite sequencing and for in vitro and in vivo experiments was separated from diseased mandarin fish in Foshan, Guangdong Province, China, in 2005 (OP896201.1)^52^. After infecting MFF-1 cells for 7 days at a multiplicity of infection (MOI) of 0.1 with virus diluted in fresh medium containing 10% FBS and a final concentration of 10 μM 5-Azacytidine (MCE, USA), samples collected by repeated freezing and thawing were centrifuged at 5000 g at 4 °C for 30 min, and the supernatant was filtered through sterile syringe filters with a 0.45 µm pore size (Millipore, USA) to obtain hypomethylated ISKNV strains (named ISKNV-Aza). The collection procedure for wild-type ISKNV strains was as described above, except that no 5-Azacytidine was added. Virus titer 50% tissue culture infectious dose (TCID_50_) assays in MFF-1 cells were analyzed by using the Reed-Muench method^53,54^. Inactivated ISKNV or inactivated ISKNV-Aza were obtained by treating ISKNV strains or ISKNV-Aza strains at 50 °C for 24 h^28^. MFF-1 cells were infected with the treated virus strains and observed continuously by microscopic examination, qPCR, and RT-qPCR to ensure that the treated virus strains had lost activity and could not replicate and transcribe in vitro. DNA, RNA, or proteins were collected from the infected cells for analysis after infection 24, 48, 72, 96, and 120 h with virus diluted in fresh medium. The collection time of each experiment is clearly shown in the figure notes.

### Virion purification

Wild-type ISKNV strains that had been repeatedly freeze-thawed^53,55^, centrifuged, and filtered were centrifuged at 28,000 rpm at 4 °C for 1 h using Optima™ XE-100 with a Type 70 Ti Fixed-Angle Titanium Rotor (Beckman Coulter, USA). The precipitates, which were collected and resuspended in phosphate-buffered saline, were centrifuged at 150,000 g at 4 °C for 1 h using an SW 41 Ti Swinging-Bucket Rotor (SW 41 Ti) after being added to the upper layer of 35% sucrose (Sigma, USA) solution. Then, the supernatant was collected and centrifuged at 200,000 g at 4 °C for 1 h using SW 41 Ti after being added to the upper layer of a discontinuous sucrose solution density gradient (30%, 40%, 50%, and 60%, from the top to the bottom). The white suspensions located between 50% and 60% sucrose solutions were collected, resuspended in PBS, and centrifuged at 150,000 g at 4 °C for 1 h using SW 41 Ti. The final white precipitates are the purified virions.

### Plasmid and small DNA fragment

DNA from purified wild-type ISKNV virions was extracted by using a DNA isolation mini kit (Vazyme, China). The full-length DNA of ISKNV DNA methyltransferase (isknv*DNMT*), which was amplified by PCR, was inserted into the pCMV-Myc-C vector (Miaoling, China) through *Eco*R I and *Kpn* I (TaKaRa, China) restriction sites to generate a Myc-tagged expression construct (named pCMV-isknv*DNMT*-Myc). The full-length DNA of ISKNV major capsid protein (isknv*MCP*), which was amplified by PCR, was inserted into the pMD^TM^19-T vector (TaKaRa, China) to generate a plasmid named pMD19-isknv*MCP*. DNA from purified viral DNA was digested by *Eco*R I and *Kpn* I, and the fragmented DNA was recovered using a gel extraction kit (Omega, USA) after electrophoresis. The recovered DNA was ligated to the pMD^TM^19-T vector (Takara, Japan), and a 388 bp DNA fragment from the ISKNV genome was isolated. The nucleotide sequence of the 388 bp DNA fragment was included in Supplementary Note 1. The small DNA fragment was amplified by PCR to obtain the unmethylated DNA fragment, and the methylated DNA fragment was obtained after treatment with CpG methyltransferase (M. Sss I, NEB, USA). All plasmids and DNA fragments were sequenced by Guangzhou Ruibio Biotechnology Co., Ltd, Guangzhou, China for confirmation. In this study, Transfect EZ 3000 Plus (eLGbio, China) was used to transfect plasmids or DNA fragments into cells at a ratio of 3 μL EZ3000 per 1 μg plasmid or DNA. The primers used are listed in Supplementary Data 3.

### Cell activity detection

Cells were cultured in 96-well plates and replaced with fresh DMEM with 10% FBS and different concentrations of 5-Azacytidine or Dimethyl sulfoxide (DMSO) after 24 h. Cell Counting Kit-8 (Dojindo, Japan) was used to detect cell activity according to the manufacturer’s instructions. After 1 or 6 days of culture, the medium in each well was replaced with 100 μL of fresh medium, and 10 μL of CCK-8 solution was added. After the cells were cultured in the original environment for 90 min, the absorption at 450 nm and 650 nm were detected, and the absorption at 450 nm minus that at 650 nm was used as a measure of cell activity.

### Methylation-specific PCR (MSP) and bisulfite sequencing PCR (BSP)

DNA from samples was extracted using a DNA isolation mini kit and processed separately using a DNA bisulfite conversion kit (Tiangen, China). The processed DNA was amplified using EpiTaq^TM^ HS (TaKaRa, China) according to the following program: 35 cycles of 98 °C for 10 s, 55 °C for 30 s, and 72 °C for 30 s. For MSP, the reaction solution was electrophoresed, and the images were acquired from Tanon^TM^ MINI Space 2000. For BSP, the processed DNA was amplified as described for MSP but with different primers. The DNA from the reaction solution was electrophoresed, recovered, and ligated to the pMD^TM^19-T vector and transformed into *E. coli* DH5α competent cells. After incubation, positive bacteria were expanded, cultured, and subjected to sequencing after plasmid extraction. The sequencing results were compared and analyzed using DNAMAN X. The primers used are listed in Supplementary Data 3.

### Absolute quantitative PCR (qPCR)

Cells were cultured in 24-well plates to assay virus replication and cultured in 6-well plates to assay virus adsorption and invasion. DNA from infected cells was extracted using a DNA isolation mini kit after repeated freezing and thawing. The copies of the ISKNV genome in the samples were quantified by using 2 × real PCR Easy Mix-TaqMan (Foregene, China) according to the standard curve, which was determined from a series of diluted pMD19-isknv*MCP* plasmids. The reaction programs were as follows: 95 °C for 1 min, followed by 45 cycles of 95 °C for 15 s, 60 °C for 30 s, and 72 °C for 1 s. The primers used are listed in Supplementary Data 3.

### Real-time quantitative PCR for reverse transcription (RT-qPCR)

Cells were cultured in 12-well plates, and each mandarin fish received 100 μL virus or an equal volume of DMEM by intraperitoneal injection. The RNA samples were reverse-transcribed to cDNA using a PrimeScript RT Reagent Kit (Takara, China) after the total RNA from transfected or infected cells or fish tissue was extracted using a total RNA extraction kit (Promega, China). RT-qPCR assays were conducted using SYBR Green Pro Taq HS (Accurate Biology, China) with a Roche LightCycler® 480 System, while *S. chuatsi β-actin* or *GAPDH* was used as the reference gene. The reaction programs were as follows: 50 °C for 2 min and 95 °C for 10 min, followed by 40 cycles of 95 °C for 15 s, 60 °C for 15 s, and 72 °C for 15 s. The primers used are listed in Supplementary Data 3.

### Western blot analysis

Cells were cultured in 6-well plates. The infected cells were collected after infection 48 and 72 h and lysed in 200 μL of Passive Lysis Buffer (Promega, USA), and the protein concentration was determined using a BCA protein quantification kit (Genesand, China). Equal amounts of protein samples were separated via SDS-PAGE and transferred to a 0.45 µm PVDF membrane (Millipore, USA). The protein expression levels of ISKNV were measured by a mouse anti-isknvORF108L (an ISKNV viral structural protein) antibody (mAb2D8, from Dr. Chuanfu Dong). The antibody was also named anti-ISKNV-VP101L. To manifest equal protein sample loading, *S. chuatsi* GAPDH expression was measured using rabbit anti-GAPDH (Abways, China). Goat anti-mouse IgG (H + L) and goat anti-rabbit IgG (H + L) (Promega, USA) were used as the secondary antibodies. Finally, the images were acquired from Amersham Image 600 using a High-sig ECL Western blotting substrate kit (Tanon, China).

### Dual-luciferase reporter assays

Cells were cultured in 24-well plates and replaced with fresh DMEM with 10% FBS before transfection. The interferon-β luciferase reporter plasmid (pGL3-*IFN*β-luc, 0.4 μg), small DNA fragment (0.5 or 1.0 μg), and pRL-TK (0.04 μg) plasmid were cotransfected into MFF-1 cells. The pRL-CMV plasmid was transfected as an internal control. After 36 h of transfection, cells were collected and lysed in 200 μL of Passive Lysis Buffer (Promega, USA), and total cell lysates were subjected to the Dual-Luciferase Reporter Gene Assay Kit (Promega, USA). Luciferase activities were measured using Glomax (Promega, USA).

### Immunofluorescence assays

Cells were cultured in 24-well plates on glass coverslips. Cells were fixed at the indicated time points using frozen formaldehyde and incubated with mouse anti-isknvORF108L and rabbit anti-5-methylcytosine (CST, USA) for 1 h at 25 °C. DyLight 488-conjugated goat anti-rabbit IgG and DyLight 594-conjugated goat anti-mouse IgG (Abbkine, China) were used as the secondary antibodies. Mounting medium with DAPI (Abcam, UK) was used for nucleic acid staining. Then, the cells were observed using a Leica LSM 880 confocal microscope.

### Detection by transmission electron microscopy

Cells were cultured in 150 mm culture dishes. Three days after infection, the cells were washed with PBS, collected with pancreatin, and fixed using frozen electron microscope stationary liquid after centrifugation. Sectioning, staining, and image taking were performed by Hangzhou Haoke Biological Co., Ltd, Hangzhou, China.

### Whole-genome bisulfite sequencing (WGBS)

DNA from purified wild-type ISKNV virions was extracted, and the obtained DNA libraries were constructed, including fragmentation, terminal repair, A-tail addition, splice addition, fragment screening, bisulfite treatment, and PCR amplification. The qualified libraries were sequenced on an Illumina NovaSeq 6000 platform (PE150). Library construction and sequencing were performed by Annoroad Gene Technology Co., Ltd, Beijing, China. The raw data were trimmed and filtered by Trimmomatic v0.39^56^ (“ILLUMINACLIP:TruSeq3-PE-2:2:30:10:8:true SLIDINGWINDOW:5:20 LEADING:5 TRAILING:5 MINLEN:36”). Clean data were mapped to the ISKNV genome, deduplicated, and methylation extracted (--ignore_r2) using Bismark v0.24.1^57^, Bowtie2 v2.5.2^58^, and SAMtools v1.17^59^. SAMtools was also used to determine the sequencing depth.

### Partition of CpG Islands (CGIs)

CGIs in the ISKNV genome were partitioned using EMBOSS Cpgplot and EMBOSS Newcpgreport in EMBL-EBI^60^.

### Protein domain analysis, structure prediction, and evolutionary tree

SMART^61^ was used for domain analysis of proteins. The division of the DNA methylation catalytic domain of DNMT was based on Pósfai, J. et al.^62^. The monomer_casp14 model in AlphaFold v2.3.1^63^ was used to predict the structure of proteins. AlphaFold’s database versions were the default except for the following: 2023-03-01 for uniprot and uniref90, 2023-03-03 for pdb_mmcif and pdb_seqres. PyMOL v2.5.0 was used to align the structure of isknvDNMT to each structure of *sc*DNMT. MEGA v11.0.13^64^ was used to align the protein sequences using ClustalW and build the evolutionary tree using the neighbor-joining method with 1000 bootstrap replicates, the Jones-Taylor-Thornton (JTT) model, and complete deletion.

### Statistics and reproducibility

Statistical analysis was performed using SPSS v27.0.1. Data were normalized and represented as the mean ± standard deviation (SD). All experiments in this study had at least three biological replicates, and each sample was tested in the same manner at least three times (unless otherwise stated). Significance between groups was evaluated by using a two-tailed/unpaired Student’s *t-*test. *p-value < 0.05 and **p-value < 0.01 were considered significant and extremely significant differences, respectively.

### Reporting summary

Further information on research design is available in the Nature Portfolio Reporting Summary linked to this article.

### Supplementary information

Supplementary Information

Description of Additional Supplementary Files

Supplementary Data 1

Supplementary Data 2

Supplementary Data 3

Supplementary Data 4

Supplementary Data 5

Reporting Summary

## Data Availability

All genomic data produced in the present project have been deposited in the NCBI GEO database under accession number GSE241500 and can be found in UCSC Browser (http://genome.ucsc.edu/s/minfei/OP896201.1). The protein structure model files by Alphafold were included in Supplementary Data 4 and the numerical source data for graphs were included in Supplementary Data 5. The uncropped and unedited blot/gel images with size markers were included in Supplementary Fig. 6, Supplementary Fig. 7, and Supplementary Fig. 8.
