# Supplementary material for: Hypermethylated genome of a fish vertebrate iridovirus ISKNV plays important roles in viral infection
Source: Commun Biol. 2024 Feb 28;7:237. doi: 10.1038/s42003-024-05919-x (PMC10899263; doi:10.1038/s42003-024-05919-x)
Supplement: Supplementary file 1 — Supplementary Information [file 42003_2024_5919_MOESM1_ESM.pdf]

Supplementary Figure 1. Composition of the ISKNV genome.

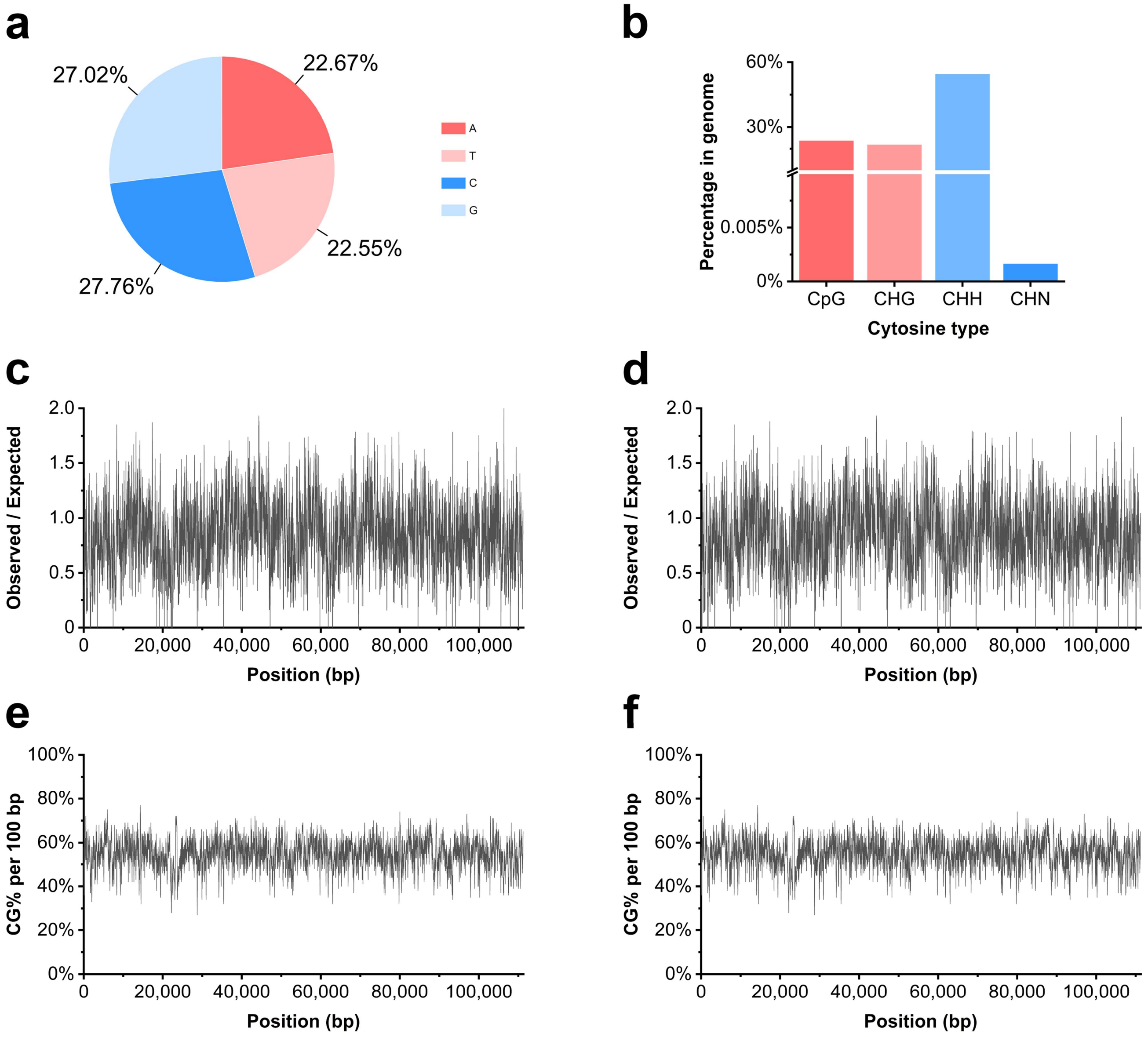

(**a**) Proportion of bases in the R strand of the ISKNV genome. (**b**) Proportion of different types of cytosines in the ISKNV genome. (**c**) Detailed data on CGI partitioning in the R strand of the ISKNV genome. (**d**) Detailed data on CGI partitioning in the L strand of the ISKNV genome. (**e**) CG% per 100 bp in the R strand of the ISKNV genome. (**f**) CG% per 100 bp in the L strand of the ISKNV genome. In the figure, "Observed" indicates the number of CpG's per 100 bp, and "Expected" indicates "(the number of C's per 100 bp × the number of G's per 100 bp) / 100".

Supplementary Figure 2. 5-Azacytidine can significantly delay the onset of symptoms in MFF-1 cells after infection with ISKNV.

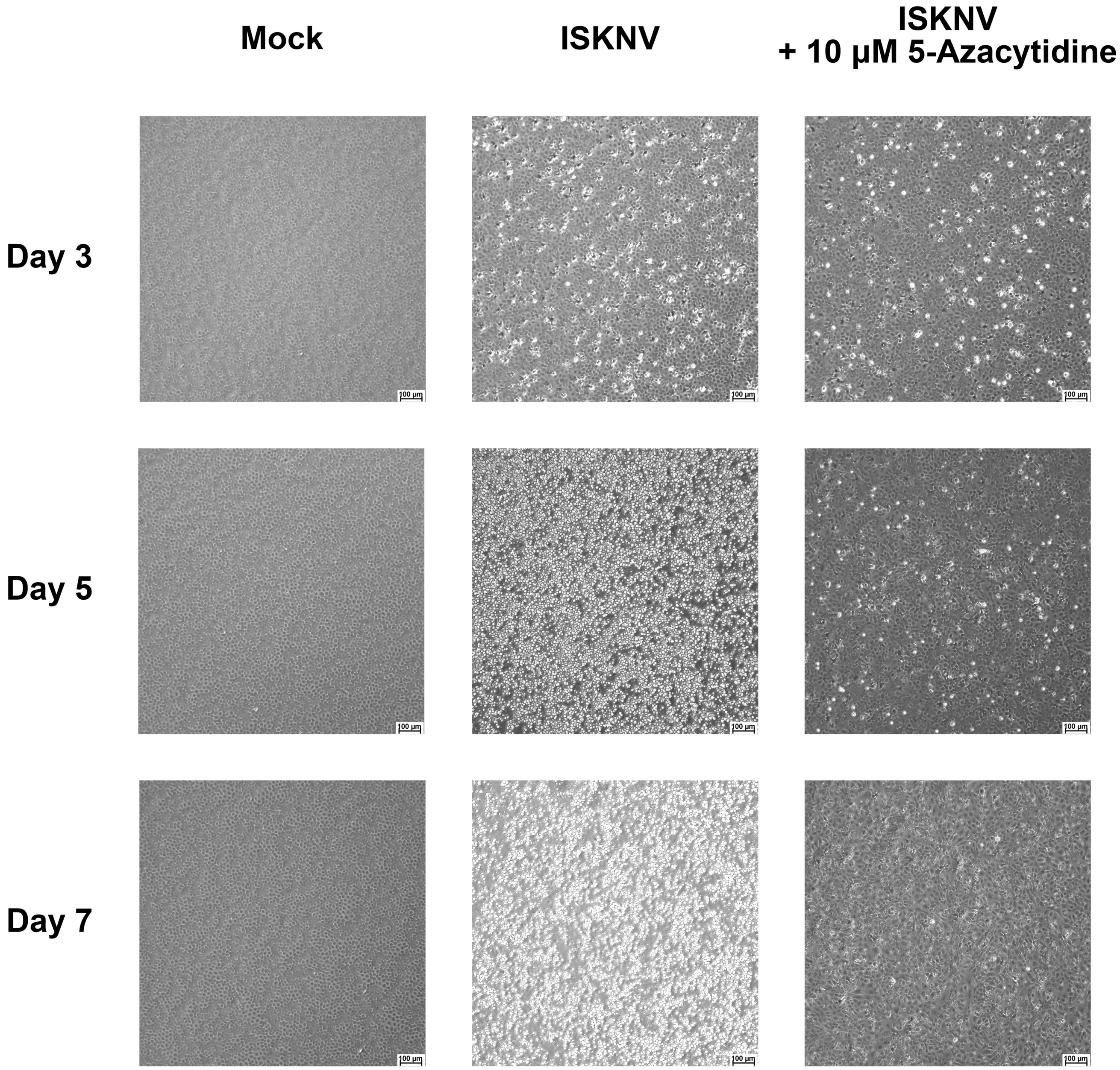

MFF-1 cells were cultured in 6-well plates and infected with ISKNV (MOI = 1) with or without 10 μM 5-Azacytidine. The scale bars in the figure represent 100 μm.

Supplementary Figure 3. The effect of 5-Azacytidine on MFF-1 cells activity.

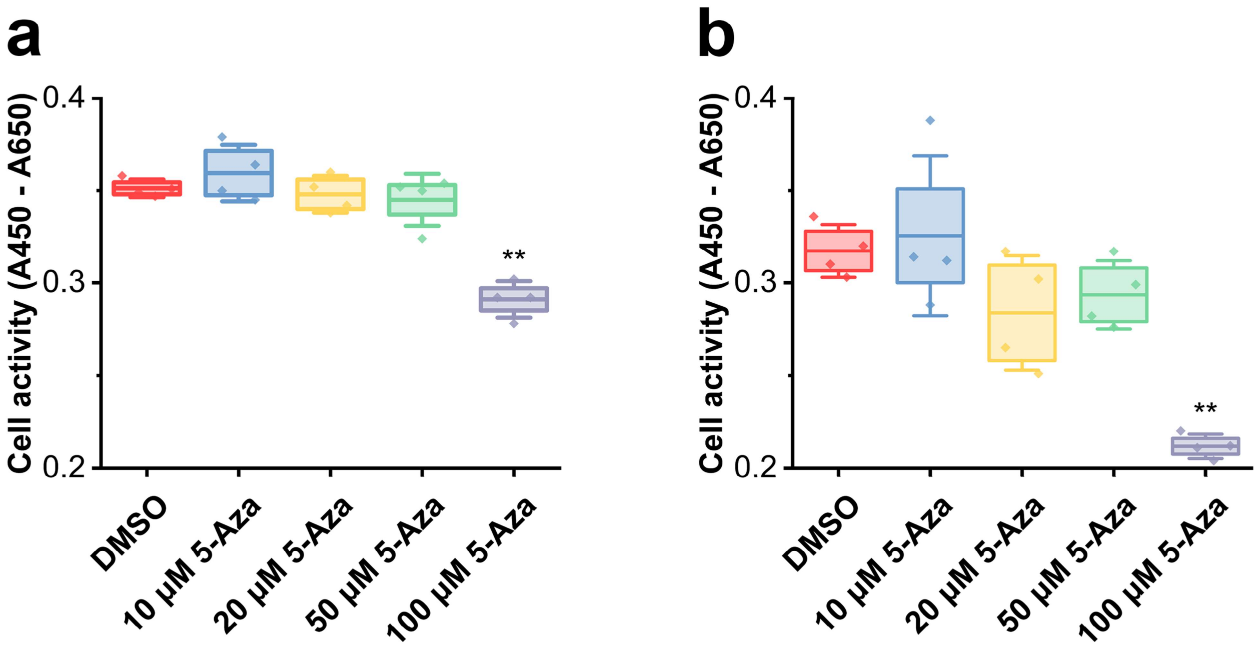

Effects of different concentrations of 5-Azacytidine on the cell activity of MFF-1 cells after 24 hours (**a**) or 144 hours (**b**) of culture (n = 4). In the figure, DMSO indicates Dimethyl sulfoxide, 5-Aza indicates 5-Azacytidine, A450 indicates the absorption at 450 nm, and A650 indicates the absorption at 650 nm. The box limited is from the upper to the lower quartiles. The center lines represent the mean and the whiskers represent the SD. Statistical significance is indicated by asterisks, with *p value <0.05 and **p value <0.01.

Supplementary Figure 4. Cell experiments proved that the virus was successfully inactivated.

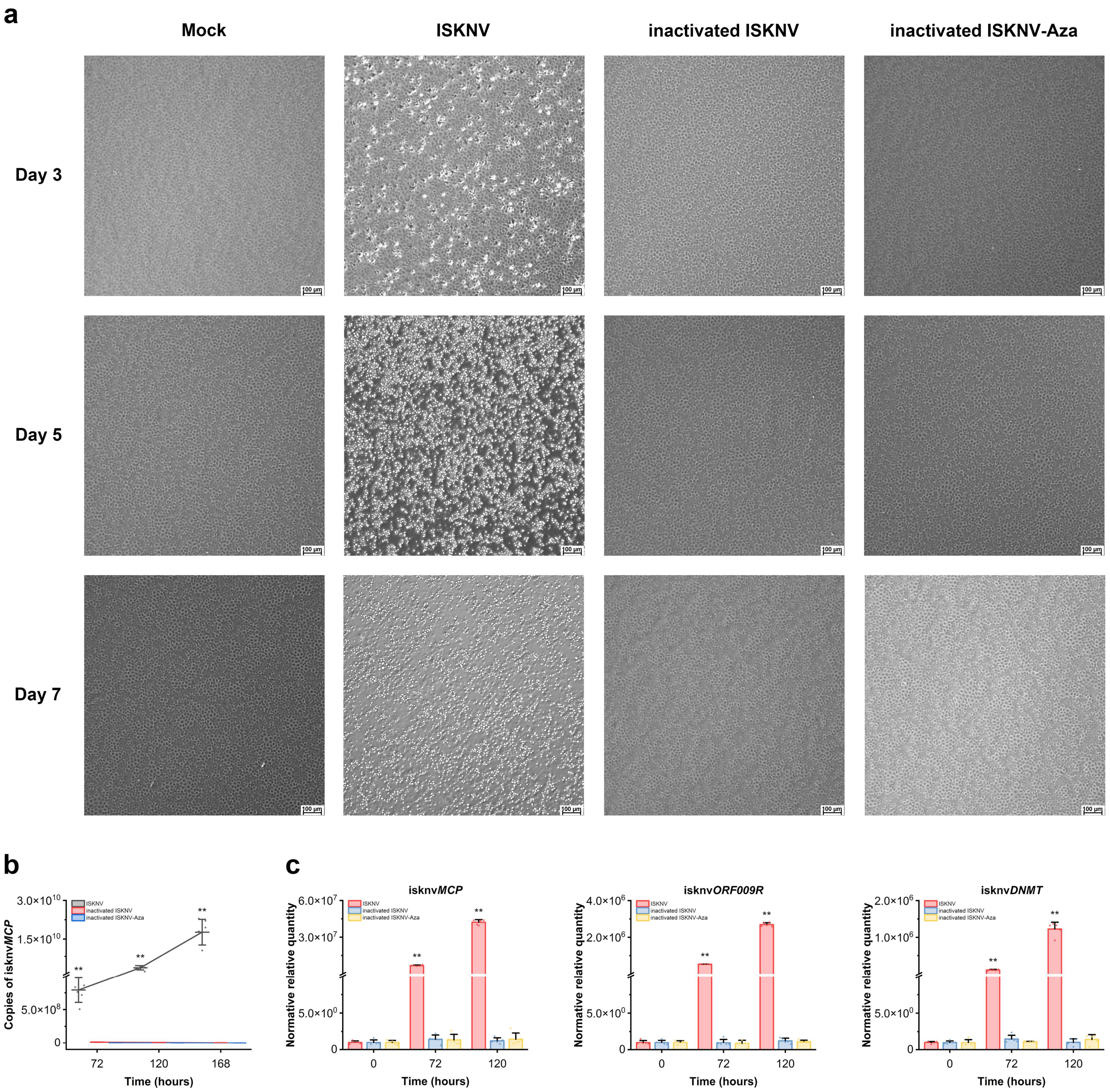

(**a**) MFF-1 cells were cultured in 6-well plates and infected with 1 x 10^9 copies/mL ISKNV, inactivated ISKNV, or inactivated ISKNV-Aza. The scale bars in the figure represent 100 μm. (**b**-**c**) MFF-1 cells were cultured in 12-well plates and infected with 1 x 10^9 copies/mL ISKNV, inactivated ISKNV, or inactivated ISKNV-Aza. (**b**) Copies of isknv*MCP* were determined by qPCR at 72, 120, and 168 hours (n = 6). (**c**) Relative mRNA levels of isknv*MCP*, isknv*ORF009R*, and isknv*DNMT* were measured by RT-qPCR at 0, 72, and 120 hours (n = 5). Data are shown as the mean ± SD. Statistical significance is indicated by asterisks, with *p value <0.05 and **p value <0.01.

Supplementary Figure 5. Prediction of the resulting protein structure confidence.

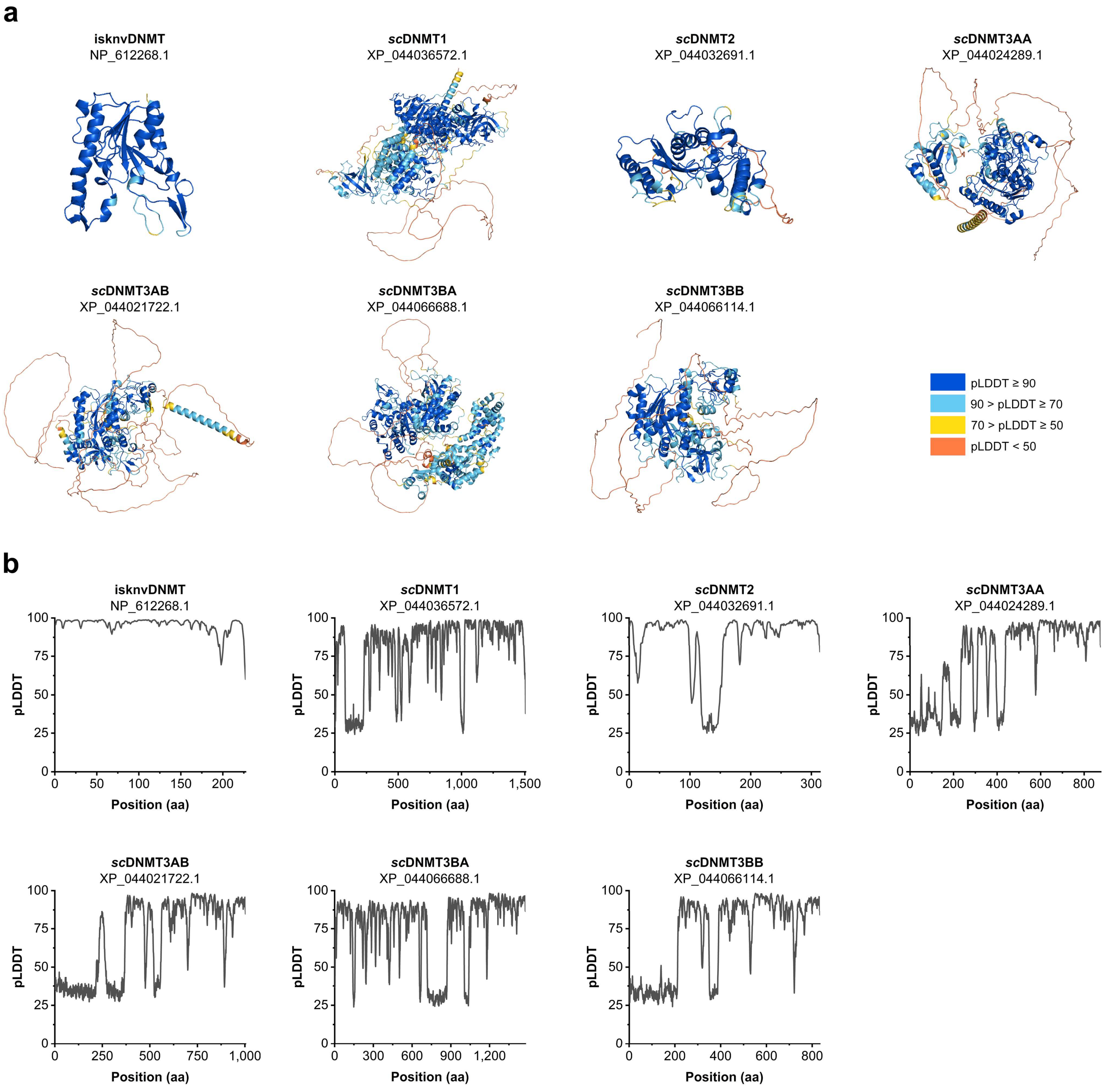

(**a**) Visualization of the confidence (pLDDT) of isknvDNMT and *sc*DNMTs in the structure. (**b**) Confidence (pLDDT) for each amino acid of the isknvDNMT and *sc*DNMTs.

Supplementary Figure 6. Uncropped and unedited gel images of Figure 3b.

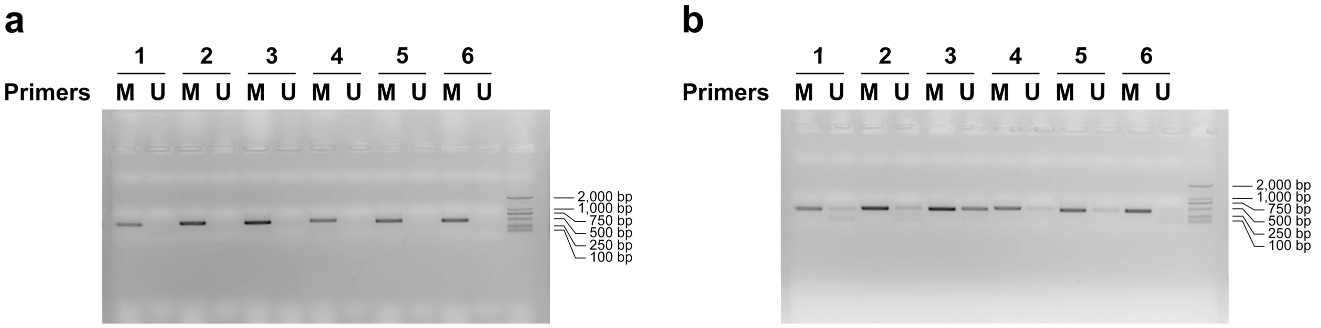

The uncropped and unedited gel images of the MSP results in the ISKNV (**a**) and ISKNV-Aza (**b**) genomes. In the figure, “M Primers” indicates methylated primers, and “U Primers” indicates unmethylated primers.

Supplementary Figure 7. Uncropped and unedited blot images of Figure 4c.

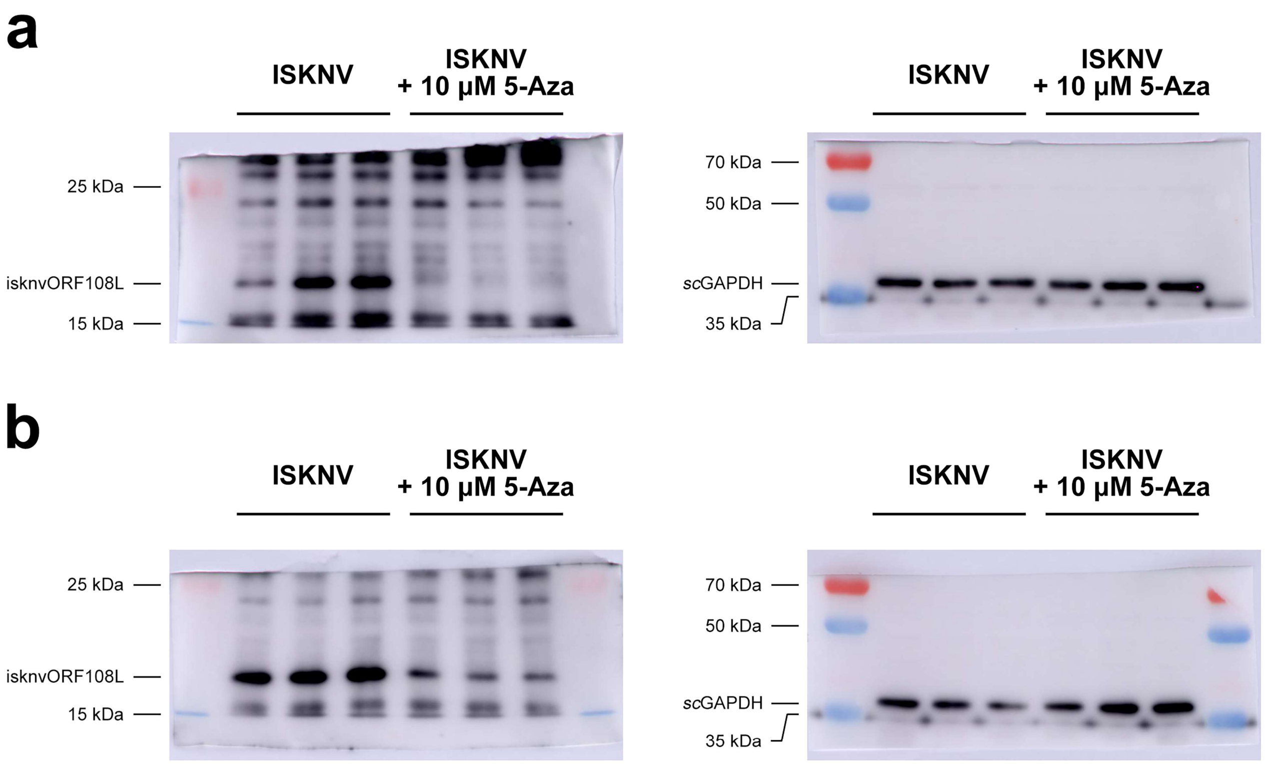

The uncropped and unedited blot images of the western blotting results at 24 hours (**a**) and 48 hours (**b**) after infecting MFF-1 cells with ISKNV with different concentrations of 5-Azacytidine. In the figure, “5-Aza” indicates 5-Azacytidine.

Supplementary Figure 8. Uncropped and unedited gel images of Figure 7d.

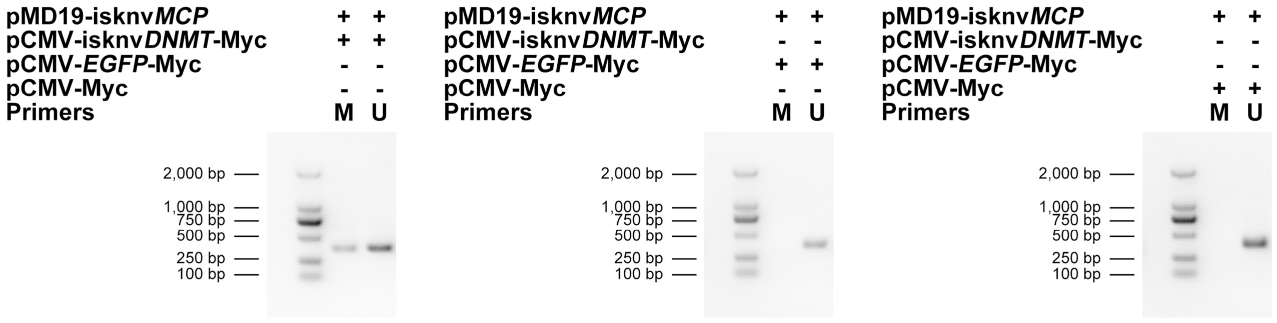

The uncropped and unedited gel images of the MSP results after transfecting different plasmids into cells. In the figure, “M Primers” indicates methylated primers, and “U Primers” indicates unmethylated primers.

Supplementary Note 1. The nucleotide sequence of the 388 bp DNA fragment from 5’ to 3’.

GTAAAGCTCGGTCGCCTTAACGGTGCCACAGCGCACAAAGGCCGCCTCGAGTGTGGCGTCT

GTGAACACCGTGCCAAAGCCCCGCCGCAACGTCTCGAGCTGCGCCATCGAGGGATGGGGAC

TGCGGCGCGTAAAGTAAGACTCCATGATTTTTATTACAAAAATGTATAGTCTCCTGGAGATTGG

CGAAGTGCTGGCCATAGGACGGCCGGACAATACAGTGTCCTTCATACACGATCGTGATAACGA

GGTCTATGTTGTCGTCCAAATTAGTGGCGGCTGCGTCCGCGTCATTGCCATGGCGGGGCAGG

ATACCCACGCGGGTAAGCTCAGGGCATGCCTTTACACTGCGGCGCACGCATACGGGCCCAAG

TTGACCATGCCGTTG
