# Supplementary material for: Hypermethylated genome of a fish vertebrate iridovirus ISKNV plays important roles in viral infection
Source: Commun Biol. 2024 Feb 28;7:237. doi: 10.1038/s42003-024-05919-x (PMC10899263; doi:10.1038/s42003-024-05919-x)
Supplement: Supplementary file 2 — Description of Additional Supplementary Files [file 42003_2024_5919_MOESM2_ESM.pdf]

Description of Additional Supplementary Files

File name: Supplementary Data 1
Description: CpG islands of ISKNV genome (OP896201.1)

File name: Supplementary Data 2
Description: 5mC methylation landscape of ISKNV genome (OP896201.1)

File name: Supplementary Data 3
Description: Primers used in this study

File name: Supplementary Data 4
Description: The protein structure model files by Alphafold

File name: Supplementary Data 5
Description: The source data behind the graphs in the paper
